# An iterative and interdisciplinary categorisation process towards FAIRer digital resources for sensitive life-sciences data

**DOI:** 10.1038/s41598-022-25278-z

**Published:** 2022-12-05

**Authors:** Romain David, Christian Ohmann, Jan-Willem Boiten, Mónica Cano Abadía, Florence Bietrix, Steve Canham, Maria Luisa Chiusano, Walter Dastrù, Arnaud Laroquette, Dario Longo, Michaela Th. Mayrhofer, Maria Panagiotopoulou, Audrey S. Richard, Sergey Goryanin, Pablo Emilio Verde

**Affiliations:** 1 European Research Infrastructure on Highly Pathogenic Agents (ERINHA), 1050 Brussels, Belgium; 2 European Clinical Research Infrastructure Network (ECRIN), 75013 Paris, France; 3 European Advanced Translational Research Infrastructure (EATRIS)/Lygature, 3521 AL Utrecht, The Netherlands; 4 Biobanking and Biomolecular Resources Research Infrastructure (BBMRI-ERIC), 8010 Graz, Austria; 5 European Infrastructure for Translational Medicine (EATRIS), 1081 HZ Amsterdam, The Netherlands; 6 European Marine Biological Resource Centre (EMBRC), University Federico II of Naples and Stazione Zoologica Anton Dohrn, 80138 Naples, Italy; 7 Department of Molecular Biotechnology and Health Sciences, Molecular Imaging Center, University of Torino, 10125 Torino, Italy; 8 European Marine Biological Resource Centre (EMBRC), 75252 Paris, France; 9 European Research Infrastructure for Biological and Biomedical Imaging (Euro-Bioimaging), Institute of Biostructures and Bioimaging. National Research Council of Italy (CNR), 10126 Torino, Italy; 10 Coordination Centre for Clinical Trials, Heinrich Heine University Düsseldorf, 40225 Düsseldorf, Nordrhein-Westfalen Germany; 11 European Clinical Research Infrastructures Network (ECRIN), Kaiserswerther Strasse 70, 40477 Düsseldorf, Germany

**Keywords:** Biochemistry, Computational biology and bioinformatics, Ecology, Genetics, Microbiology, Molecular biology, Plant sciences, Zoology, Ecology, Natural hazards, Medical research

## Abstract

For life science infrastructures, sensitive data generate an additional layer of complexity. Cross-domain categorisation and discovery of digital resources related to sensitive data presents major interoperability challenges. To support this FAIRification process, a toolbox demonstrator aiming at support for discovery of digital objects related to sensitive data (e.g., regulations, guidelines, best practice, tools) has been developed. The toolbox is based upon a categorisation system developed and harmonised across a cluster of 6 life science research infrastructures. Three different versions were built, tested by subsequent pilot studies, finally leading to a system with 7 main categories (sensitive data type, resource type, research field, data type, stage in data sharing life cycle, geographical scope, specific topics). 109 resources attached with the tags in pilot study 3 were used as the initial content for the toolbox demonstrator, a software tool allowing searching of digital objects linked to sensitive data with filtering based upon the categorisation system. Important next steps are a broad evaluation of the usability and user-friendliness of the toolbox, extension to more resources, broader adoption by different life-science communities, and a long-term vision for maintenance and sustainability.

## Introduction

A succession of global challenges (e.g., climate change, epidemics, loss of biodiversity, resource scarcity, economic dislocation) has underlined the need to pool data and digital resources from the life sciences (LS). A high proportion, however, of the data generated by LS research can be considered sensitive, and this is sometimes used as an argument against data sharing and harmonisation. The development of disciplinary vocabularies and ontologies, coming from Artificial Intelligence (AI) laboratories, has been encouraged and justified for a long time^1,2^, and their creation is now well documented, particularly in the biomedical field (e.g.,^3^. Regarding sensitive data in the broader field of LS, an agreed vocabulary should improve a common classification of resources relating to this sensitivity. Even if the creation of vocabularies, encouraged since 2016 by the publication of the FAIR principles^4^, is exponential, as shown by general catalogues (e.g. Metadata Standards Catalog (MSC) maintained by the Metadata Standard Catalog RDA WG^5^ or Schema.org, https://schema.org/) or disciplinary ontology portals (e.g. Bioportal, Agroportal, and more recently Ecoportal, through the Ontoportal alliance (https://ontoportal.org/), and facilitated by free and well recognized tools (e.g. prot, https://protege.stanford.edu/), the subject of sensitive data is poorly treated, its cross-disciplinary nature and dependence on national legislation making it more difficult to understand. Such data can include most of the categories of sensitive data defined by the Sensitive Data Interest Group of the Research Data Alliance (RDA), including personal data, environmental data, proprietary data, Dual Use Research of Concern (DURC) data and classified information (https://www.rd-alliance.org/groups/sensitive-data-interest-group)^6^. For LS research infrastructures (RIs), this creates an additional layer of complexity on top of the more generic technical and data security issues linked to data sharing.

Cross-domain categorisation and discovery of digital resources related to management of sensitive data presents major challenges. In 2016, Wilkinson et al. provided 15 FAIR principles intended to promote Findability, Accessibility, Interoperability and Reusability of data, even when they are not openly available^4^. Several of these principles require common vocabularies and sufficient, appropriate and community approved transdisciplinary metadata to achieve global interoperability in an interdisciplinary context^7^. This needs common, sufficient, and appropriate metadata, common FAIR vocabularies links with other metadata, multiple and efficient attributes for (meta) data and resources and links to communities’ standards (see F2, I2, I3, R1, R1.3 of the FAIR principles in Supplementary, Table S1: FAIR Guiding principles). Even if AI and Machine Learning (ML) studies are also considering FAIR qualities of terms for common validated vocabularies (e.g.,^8^), inconsistencies between uses of terms need human achieved steps to validate common definitions^9^ and FAIRness compliance checking for vocabularies^10^. To achieve compliance with these FAIR principles, discrepancies between RIs must be identified and a common language must be community agreed.

To support this process, a toolbox has been developed, containing links to digital objects relating to sensitive data. Searching the toolbox makes use of a categorisation system developed and harmonised across a cluster of LS RIs. The work was performed within the EOSC (European Open Science Cloud)-Life project, bringing together the 13 Life Science ‘ESFRI’ (European Strategy Forum on Research Infrastructures) research infrastructures to create an open, digital, and collaborative space for biological and medical research (https://www.eosc-life.eu/). The objective of the toolbox is to help researchers find resources that can help them make their sensitive data available for re-use. The content of the toolbox should cover existing recommendations, procedures, best practices, and links to software (tools) to support data sharing and re-use relevant for sensitive data management. In addition, guidelines and other useful resources drafted in the context of EOSC-life will be included. The toolbox demonstrator does not contain de novo* information*; instead, it helps scientists to navigate through previously collected best quality content available throughout the EOSC-Life collective infrastructure landscape. The design of the toolbox demonstrator and its subsequent population with content is designed to be driven by use cases, stemming from within EOSC-Life, to achieve our objectives and, work was and will be done in an iterative fashion using community approval processes^6^.

## Methods

In the development of the categorisation system and the toolbox the LS RIs listed in Table 1 were involved. Table 1 also contains a short definition of RIs and a description of cluster projects.

**Table 1:** Involved Research Infrastructures from the Life Sciences and definition of research infrastructures and cluster projects.

**Research Infrastructure (RI)**	
Definition of RIs	The European Commission (EC) is defining Research Infrastructures (RIs) as facilities that provide resources and services for research communities to conduct research and foster innovation. They can be used beyond research e.g., for education or public services and they may be single-sited, distributed, or virtual. They often include: i) major scientific equipment or sets of instruments, ii) collections, archives or scientific data, iii) computing systems and communication networks, iv) any other research and innovation infrastructure of a unique nature that is open to external users
Cluster projects with RIs	The European Strategy Forum on Research Infrastructures (ESFRI, https://www.esfri.eu/) was established by the EC to coordinate policy-making on Research Infrastructures in Europe. It identified five thematic areas to pave the way for Open Access data for the European Open Science Cloud and the following five “cluster projects” were funded: 1) EOSC-Life (https://www.eosc-life.eu/) bringing together the Biological and Medical RIs, 2) ENVRI-FAIR (https://envri.eu/home-envri-fair/) on environmental research, 3) ESCAPE (https://projectescape.eu/) in the area of astronomy- and accelerator-based particle physics, 4) SSHOC (https://www.sshopencloud.eu/) in the social sciences and humanities area, 5) PaNOSC (https://www.panosc.eu/) in the field of Photon and Neutron science
**Research Infrastructures involved in the project**	
Abbreviation	Name
BBMRI	Biobanking and Biomolecular Resources Research Infrastructure
EATRIS	European Advanced Translational Research Infrastructure in Medicine
ECRIN	European Clinical Research Infrastructure Network
EMBRC	European Marine Biological Resource Centre
ERINHA	European Research Infrastructure on Highly Pathogenic Agents
Euro-Bioimaging	European Research Infrastructure for Biological and Biomedical Imaging

A concept description of the toolbox was provided at an early stage of the project^11^.

### Categorisation system and pilot studies

**Figure 1:**
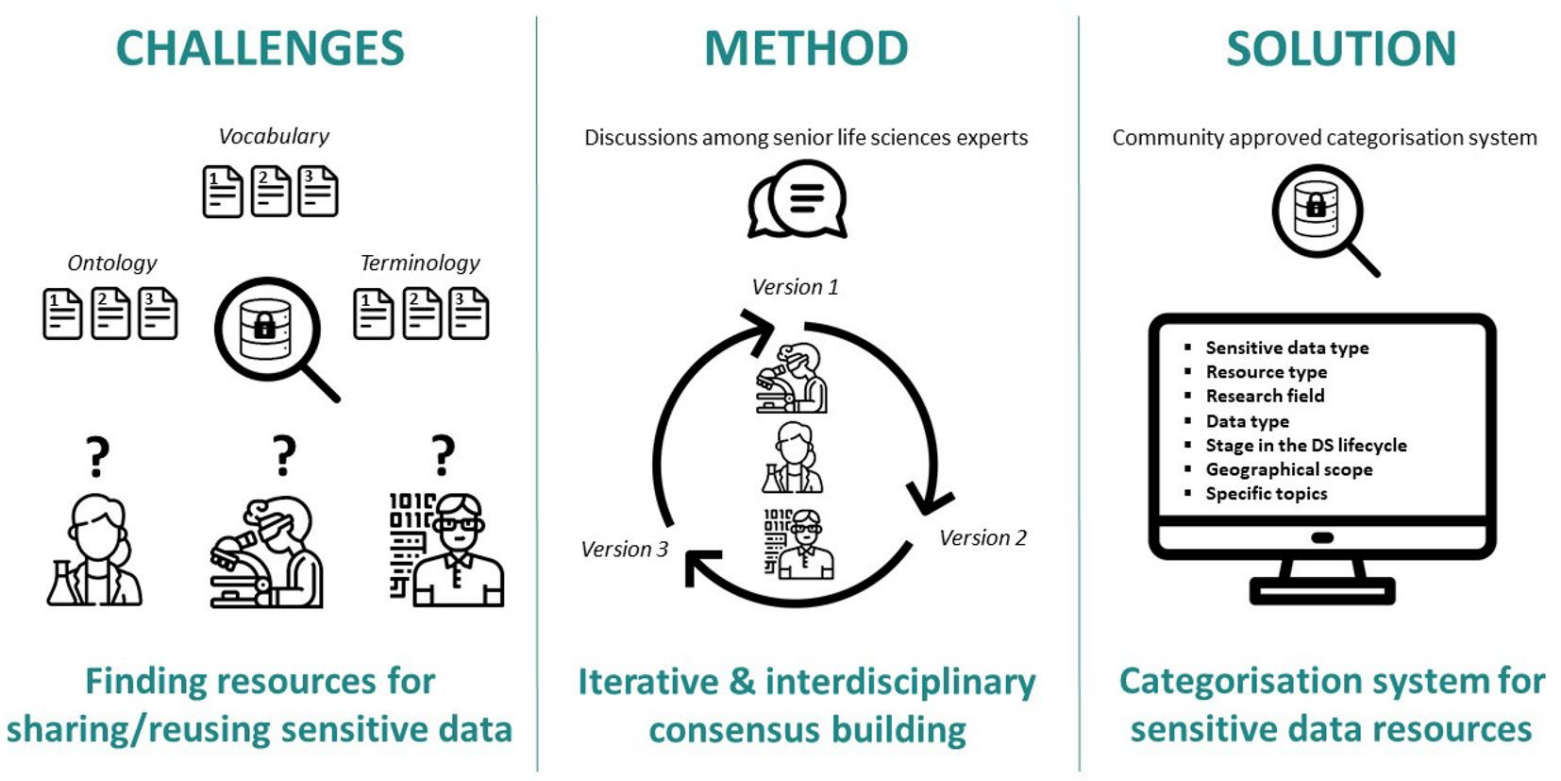
Graphical abstract: Challenges, method and solution.

Three different versions of the categorisation system were developed, each tested by a subsequent pilot study, and all published in Zenodo^12–14^, as illustrated in Fig. 1. To mitigate polysemic issues and to improve applicability and understandability, the different categories, and the individual tags of the categorisation system to be constructed were discussed in regular meetings of a multidisciplinary working group (WG) covering various RIs, and common definitions were adopted across RIs.

Existing terminologies, vocabularies and ontologies were used as a start to develop the version 1 of the categorisation system (see Table 2). Unfortunately, none of the existing resources was perfectly fitting the scope of the project (e.g., not RDA MSC^5^, EDAM^15^, BioPortal^16^).

**Table 2:** Terminologies, vocabularies and ontologies used in the development of the categorisation system.

Category	Existing terminologies, vocabularies or ontologies used for the categorisation system
Sensitive data type	RDA SHARC interest Group^6^ NIH, Office of Science Policy: Dual Use Research of Concern^17^ NIST, Information Technology Laboratory. Computer Security Resource Center. Glossary: Classified information^18^
Resource type	For specifying the category “Resource type”, no formal vocabulary or ontology was taken into consideration. Starting from specific use-cases in EOSC-Life, the group developing the system, identified and agreed a list of “Resource types” most relevant for the topics under discussion and the content of the toolbox
Research field	OECD^19^ Cambridge Author Services: Areas of Study^20^ ANZSRC, National Health and Medical Research Council Fields for Research (FOR)^21^ The World University Rankings: Subject Ranking 2015–2016: life sciences top 100^22^ Clarivate, Web of Science Core Collection Help: Research Areas (Categories/classification)^23^ EDAM Ontology of bioscientific data analysis and data management^15^ The different sources were analysed for suitability to the project and compared and the group agreed a final list
Data type	As a starting point, the definition of “Data type” was based on feedback from the participating RIs, indicating data types used in their scientific domain. The feedback was structured and aligned into broad categories by the group, differentiating into data from living humans and data not derived from living humans
Stage in data sharing life cycle	UK Data Archive: Data life cycle & data management planning^24^ EDAM ontology of bioscientific data analysis and data management^15^ Classification of processes involved in sharing individual participant data from clinical trials^25^ In version 1 of the categorisation system a more detailed list of stages was included, which had to be simplified in the latter versions due to application issues reported from the pilot studies
Geographical scope	UN: Standard country or area codes for statistical use^26^
Specific topics	RDA CASRAI research data management glossary^27^ CODATA research management glossary^28^ In addition, the list of “Specific topics" originated from the issues and use cases raised in the EOSC-Life project

Here only the categories from version 3 are considered. In version 1 two more categories “research design ^29–34^ and “targeted group [35] were considered but not included in the final version 3.

In the field of sensitive data, there is no specialised terminology/vocabulary/ontology; often all sciences are covered but not specifically the field of sensitive data in the LS. From the sources available, we took out elements, which seemed to be useful for the individual dimensions of the categorisation system. Ideas were taken up and put together and proposals were developed, which categories to include and which not. To come forward we first had to agree on the terminology and to come to a common understanding. This work was done among the experts of the 6 RIs. The next step will be the alignment of the work with existing classifications and exploration with a larger audience and other RIs from LS.

For evaluation of the first categorisation system (pilot study 1), each involved RI nominated two senior experts, willing to perform the assessment. The experts selected 110 resources about sensitive data, spanning a wide range of resource types (e.g., legislation & regulations, position papers, policies and principles, background & explanatory material, recommended practice, systems, tools & services). The two experts assessed the same resources independently of each other and documented the results using the Zotero bibliographic system (https://www.zotero.org/). After assessment, the data were analysed by an independent statistician with respect to agreement/disagreement between the two experts from one RI (kappa statistics with R version 4.0.2) as well as the variation between experts from different RIs. For this exercise, a study protocol was developed and published before the start of the first pilot study^36^. Based on the results of this first tagging exercise (pilot study 1) and additional feedback from the RI experts, major gaps in the categorisation system were identified and after intensive discussions, a simplified and revised second version of the categorisation system was developed and adopted by the WG^13^. The re-tagging exercise with the second version of the categorisation system was performed for the same 110 resources (pilot study 2). This time only one expert from each of the 6 RIs was involved, selected from the experts involved in pilot study 1. The re-tagging (pilot study 2) was performed with a web-based software tool developed by ECRIN. The data were analysed with R to determine the variation of tagging between the RIs. The subsequent re-tagging exercise (pilot study 2) still highlighted practical shortcomings, which led to the decision to revise the categorisation system. Finally, the same resources were re–re-tagged (pilot study 3) with categorisation system version 3 by the same experts as involved in pilot study 2 and a similar analysis was performed.

The concept behind the categorisation system is clarity and simplicity. For that reason and from version 2 on, clear definitions for all categories and tags were provided to support the tagging experts in their assessment process. The terms and definitions were discussed among all participating RIs at regular remote video conferences. Terminology and definitions were adapted or extended if necessary. In this process generalisation to LS RIs was elaborated and agreement was achieved to come to a prototype. The results achieved in this project must be applied in future work and tested by more users to come to more mature definitions. The categories are orthogonal in the sense that no item identified in a resource is a member of more than one category, that is, the categories are mutually exclusive. In addition, for some of the categories only one tag is usually expected (e.g., “sensitive data type”, “resource type”, “research field”, “geographical scope”). Even where multiple tags are possible, the number should be as low as possible. A logistic regression was used to model the number of resources where only one tag was used per category as dependent variable. In this model we used the pilot study number and the categories as fixed effects. Additionally, a logistic regression correcting for overdispersion was applied and the results were presented as estimated coefficients and a 90% and 95% confidence interval. The statistical methodology is described in Supplementary S5.

## Results

### Categorisation system

The changes in the different versions of the categorisation system because of the evaluation studies are presented in Supplementary Table S2 (Categories and pre-listed tags for the three different versions of the categorisation system). The initial version of the categorisation system covered 8 categories (resource type, research field, research design, data type, stage in data sharing life cycle, geographical scope, specific topics, perspective) with 93 pre-listed tags. The results of this pilot study 1 are published in^37^ and summarised here. The inter-observer reliability analysis between the two experts from one RI demonstrated very high agreement for the two experts from BBMRI (median kappa for 25 resources: 0.84). The kappa values were much lower for the other RIs with a range between 0.44 for ECRIN and 0.22 for EMBRC (median kappa value). A wide variation of the number of times a given tag was assigned to a resource in the assessment process was observed per RI and in total. The analysis revealed the following issues: missing standard definitions, no training beforehand, unclear application of some of the categories (e.g., perspective, geographical scope), inconsistent application of the tags (e.g., high variation in the number of tags per resource), missing pre-listed tags and no precise criteria for the selection of the resources. It was therefore decided to simplify the categorisation system, to make it more intuitive and to provide a glossary with definitions. In the updated categorisation system version 2, two categories were dropped. “Research design” was deleted because it was often not applicable, and “perspective” proved difficult to interpret and did not differentiate resources very well. The number of pre-listed tags for version 2 was reduced to 55.

Categorisation system 2 was evaluated in pilot study 2 with the same 110 resources and one expert per RI. Apart from the tagging of the resources, additional feedback was received from five RIs, mainly related to the content and structure of the categorisation system. The category “data type” was seen as too complex and created major discussion. Several experts strongly suggested to better define this category and to make it easier to use. This input was accommodated in the next generation of the categorisation system (version 3). The “data type” dimension was clarified by the addition of a new "sensitive data type" category, listing the main types of sensitive data. This also allowed the number of tags in "specific topics" to be reduced. Thus, categorisation system version 3 covers 7 categories (sensitive data type, resource type, research field, data type, stage in data sharing life cycle, geographical scope, and specific topics with 65 tags (see Table 3). In the Zenodo publication of version 3 agreed definitions for the categories and tags are included^14^.

**Table 3:** Categorisation system version 3 (short version) ^14^.

Categorisation system (short form, version 3, final, 26 October 2021) ^14^						
0	1	2	3	4	5	6
Sensitive data type (n = 6)	Resource type (n = 5)	Research field (n = 12)	Data type (n = 18)	Stage in DS life cycle (n = 6)	Geographical scope (n = 5)	Specific topics (n = 13)
Personal data Environmental data Proprietary data DURC data Classified information Other sensitive data	Legislation and regulations Guidelines, recommendations and policies Descriptions of (best) practice Support systems and tools Other (background) material	Any research field Clinical research Biomedical research Cell biology, molecular biology, and biochemistry Plant and mycological sciences Zoology Microbiology Marine/ water biology Ecology Environmental sciences Life science—other topics Social sciences, legal or ethical research	Data about or from living humans: Any type of data Population level health/or socio-economic data Real world or routine health data Clinical research data Public health emergency data Biobank and sample data Data with images of humans Omics and related data (from living humans) Other specific data type Data not derived from living humans: Any type of data Population level data Real world Observational/interventional research Public health emergency data Biobank and sample data Data with images Omics and related data Other specific data type	Not applicable Whole cycle Primary data registration Preparation and planning for data sharing Actions at the end of a study Managing data access and requests for data re-use	Not applicable Global Continental Country groupings National	No specific topic Agreements Attribution and credit COVID-19 Data Repositories Data sharing committees FAIR and FAIRification GDPR IP-aspects/ licences Legal basis for data sharing Metadata supporting data sharing Privacy protection Research participants involvement

Categorisation system 3 was evaluated in pilot study 3 with the same 110 resources and the same experts as in the second exercise. For 1 resource there was missing data, so 109 resources could be analysed. Pilot study 3 was performed with a computer tool developed by ECRIN (SG). In general, the computer tagging tool was characterised as user-friendly and efficient, though some suggestions were made to improve functionality. One expert argued that the tagging should be done principally by one trained person or by a permanent small board, conceding however, that this may be too resource-intensive. Finally, some comments related to the selection of resources were received, e.g., to exclude resources located behind paywalls. The categorisation system version 3 raised no major concerns, indicating that after 3 cycles of improvement the system had converted to a stable state, apart from some relatively minor points (Table 3 and Supplementary table S2).

#### Tagging of resources with categorisation system version 3 (pilot study 3)

**Figure 2:**
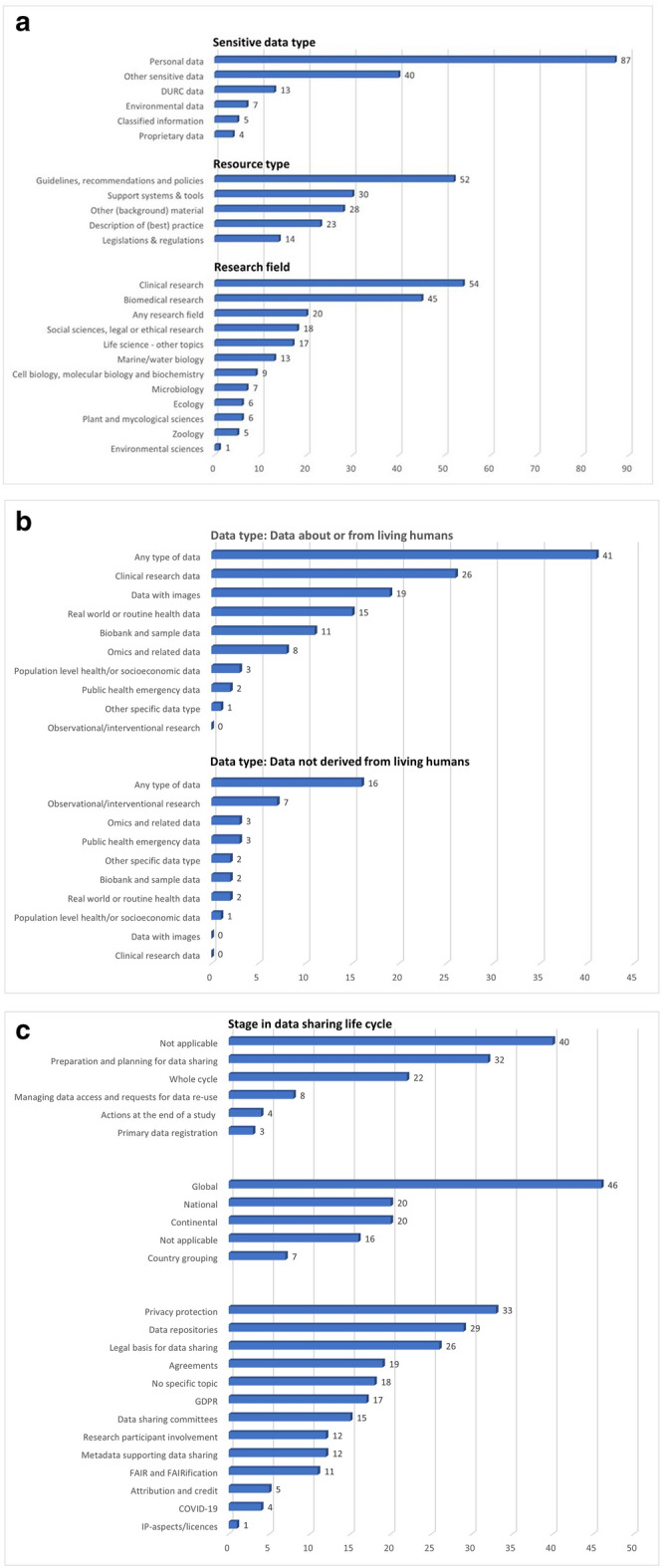
(**a**) Number of times a pre-listed tag was used for the categories « sensitive data type », « resource type » and « research field » (version 3) (**b**) Number of times a pre-listed tag was used for the category « data type » (version 3) (**c**) Number of times a pre-listed tag was used for the categories “stage in data sharing life-cycle”, “geographical scope” and “specific topics” (version 3).

The number of times a pre-listed tag was used in the assessment of the 110 resources, is presented in Fig. 2a–c.

Most resources (80%) were tagged as “personal data”. For more than one third of the resources (37%) “other sensitive data” was selected, indicating that the pre-specified list of tags needs to be extended to allow better coverage. All tags for “resource type” were used at least 10 times. For “research field” the tags “clinical research” (50%) and “biomedical research” (41%) were most often selected. In 16% of the resources no tag from the pre-specified list for “research field” could be applied and “life science – other topics” was allocated.

Data about or from living humans were the subject of most resources (82%). In this subgroup (n = 89), “any to type of data” was selected most often (n = 41). If resources were related to data not derived from living humans (n = 20), “any type of data” was also indicated most frequently (80%).

For a considerable number of resources, “not applicable” (37%) was selected to characterise “stage in data sharing life-cycle”. Most tags from the categories “geographical scope” and “specific points” were used at least 10 times.

**Figure 3:**
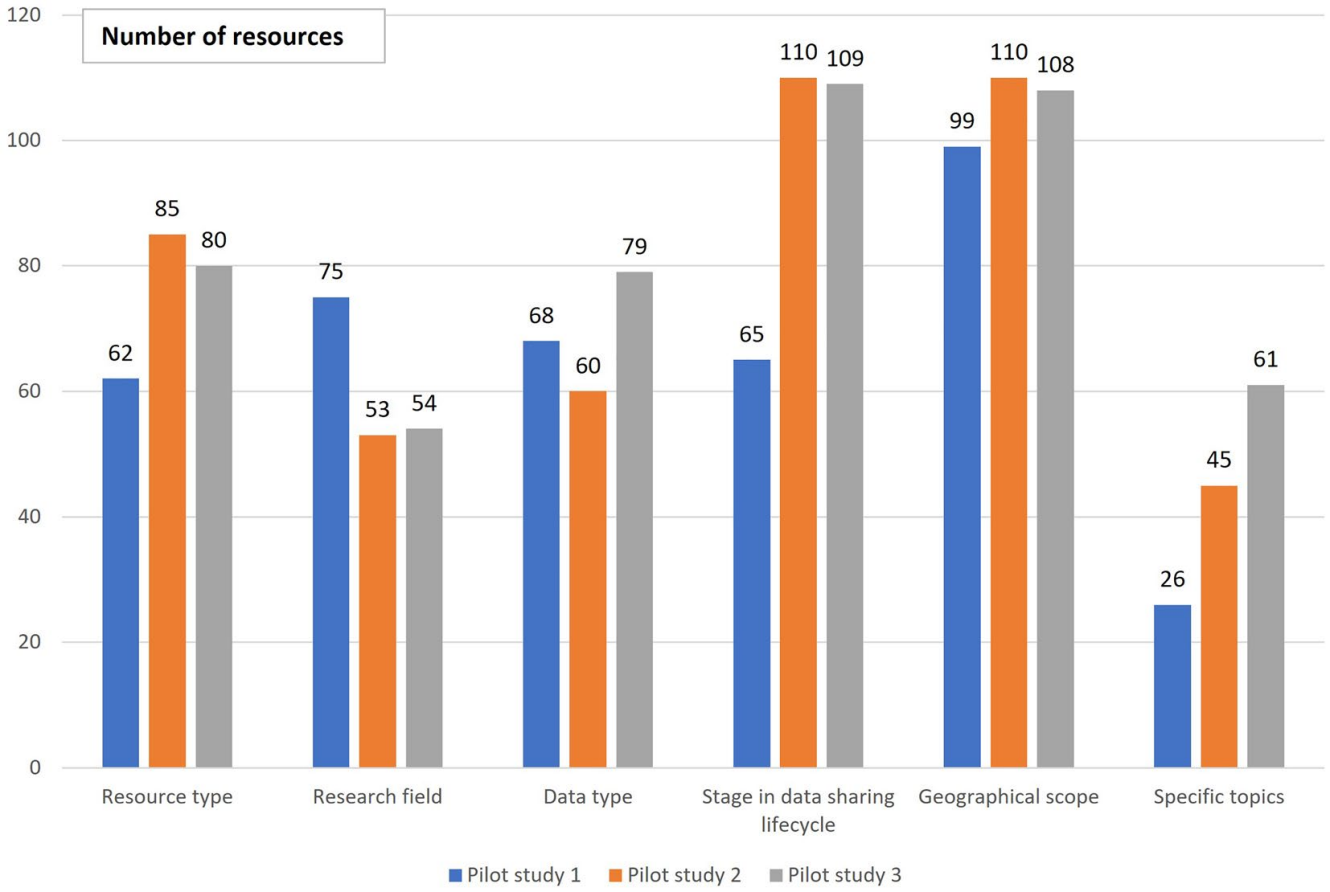
Number of resources tagged with only one tag per category for the categories applied in all three pilot studies.

In addition, the number of resources tagged with only one tag per category was analysed and the results are presented in Fig. 3 for those categories that were considered in all three pilot studies. For this analysis and the initial tagging (pilot study 1) only those experts from the 6 RIs were included that also were involved later in the re-tagging and re–re-tagging exercise (pilot study 2 and 3).

From pilot study 1 to pilot study 2 an increase of the number of resources, where only one tag per category was selected in the assessment of a resource, was observed for “resource type”, “stage in data life cycle” and “geographical scope”. For “data type” there was a slight decrease between pilot study 1 and 2 but an increase in pilot study 3. For “specific points”, there was a stepwise increase from pilot study 1 to pilot study 3. In summary, except for “research field”, the number of resources, where only one tag was allocated per category, increased over the versions. This was confirmed by statistical analysis. The coefficient for pilot study 3 versus 1 was 0.8019 with a 90% confidence interval of 0.0556 to 1.5713 and a 95% confidence interval of − 0.0861 to 1.7227, indicating a trend for the increase of the number of resources tagged only once per category from pilot study 1 to pilot study 3 (see Supplementary S5).

#### Toolbox demonstrator

##### A toolbox demonstrator was developed according to specified software requirements

The 109 resources attached with the tags from pilot study 3 were used as the initial content for the toolbox demonstrator (0ne with missing data). The toolbox demonstrator is publicly available via the link: https://tsdo.ecrin-rms.org/. The tool allows pre-filtering of resources linked to sensitive data with free text in the title, by DOI or through authors. Further filtering is possible with respect to item type (e.g., journal article, webinar, report, software) and selection of any of the pre-listed tags from the different categories of system version 3. The search result can be saved as PDF or JSON. Details of software development and implementation are summarised in the supplementary material (S6). This includes a first preliminary evaluation of the usability of the toolbox by untrained users.

## Discussion

The categorisation system was developed through an iterative procedure including a careful evaluation at each stage. This was necessary because each of three rounds yielded substantial feedback from the expert taggers, identifying issues to be resolved and proposing improvements to the system. This process led to a much clearer understanding of the structure of sensitive data resources and a wider agreement on definitions to be applied in the tagging process. In addition, the allocation of exactly one tag per category improved during the development for many categories, indicating that the selection process was straightforward for most resources and categories. As a result, the categorisation system could be simplified and the structure improved, appropriately representing a trans-disciplinary effort. This may also be important from the user perspective. At the end of the day, the system should be so intuitive that the users searching for terms would have the same logic as the experts entered the tags.

To be beneficial for the domain of LS, the categorisation system and the toolbox requires broad community approval^38,39^. In the project, we began the approval process with nominated experts from 6 LS RIs, embedded in a larger working group of the H2020-funded project EOSC-Life, covering 13 LS RIs. Though this can be seen as a useful starting point, the toolbox obviously needs community approval at a much larger scale. As the categorisation system is specifying a part of essential metadata for resources about sensitive data, it will be relevant to the FAIR Digital Objects (FDO) Forum for a « resources in the life sciences » FDO. The categorisation system can be used to derive FDO attributes and values for such FDOs. FDOs for the sensitive data itself, when levels of sensitivity and specific access protocols need to be specified is an interesting possible extension, and the categorisation system could support as a backbone information for access governance and technical choices. FDOs are to be "machine actionable", so desirable mappings between different categorisation systems will be operationalisable. New European projects such as FAIRCORE4EOSC (https://faircore4eosc.eu/), FAIR-IMPACT (https://fair-impact.eu/) and other projects working on pragmatic semantic improvements for FAIR appliance will provide possibilities for registering metadata schemas and mappings that should reuse interdisciplinary approaches in the heterogeneous field of life sciences.

The RDA has established and is maintaining a Metadata Standards Catalogue (MSC) (https://rdamsc.bath.ac.uk/mapping-index,^5^). An appropriate goal for the categorisation system would be to be included in this catalogue, after further refinement and alignment with other vocabularies addressing sensitive data in the life sciences. In any case, the work on the categorisation system can contribute to discussions on methodologies for aligning metadata schemas across scientific domains, while the categorisation system itself can be seen as an important contribution to the process of developing the most useful and appropriate cross-disciplinary terms and categories for describing sensitive data. We keep in mind that similar approaches have been applied via long and iterative processes in other scientific domains, such as understanding and predicting the evolution of climate (essential climate variables, https://public.wmo.int/en/programmes/global-climate-observing-system/essential-climate-variables) and essential biodiversity variables for mapping and monitoring species populations^40^. There are biases and gaps in the existing system that need to be tackled in the future. The initial content of the toolbox demonstrator, consisting of 110 resources related to sensitive data, has been primarily selected by four RIs with a focus on clinical and biomedical research (BBMRI, EATRIS, ECRIN, Euro-Bioimaging). Other areas and sensitive data types, such as environmental, classified, and proprietary data are under-represented, as are some disciplines, such as zoology, ecology, plant and mycological sciences, and microbiology. This indicates a need for a broader coverage of resources linked to sensitive data in the future work. Another question that needs to be investigated is how interoperable the categorisation system is with other domains outside the LS that systematically deal with sensitive data, for example, the Social Science and Humanities^41^). In addition, systematic data on the usability/user-friendliness of the toolbox from a broad sample of potential users from the life sciences are needed. Initial and informal evaluation of these aspects by the experts involved so far has been very positive but is clearly limited in scale and needs to be supplemented by more evidence.

There are major challenges to the sharing of sensitive data, including interoperability, accessibility, and governance. The primary objective of the toolbox is to improve discoverability of resources and digital objects linked to the sharing and re-use of sensitive data (F in FAIR)^4^. The systematic application of a standardised typology for resources about sensitive data, as defined by the categorisation system, helps to better structure, and organise the issues and results in metadata enrichment (F4, R1.3 of the FAIR principles in Supplementary, Table S1). The toolbox alone will not be enough for the ‘I’ of the FAIR principles, but it may become a useful backbone for building more interoperable classification systems for sensitive data resources.

It is perhaps more common to base findability on a tagging system using keywords (plus title text). That is, for example, how PubMed works—it does not categorise resources, it adds MESH terms to them (https://pubmed.ncbi.nlm.nih.gov/). Another option would have been to try to derive keywords from text or title. In our case, a categorisation system with pre-defined dimensions and pre-listed tags was preferred by the expert group. Keywords, in isolation, suffer from several disadvantages:

- A range of equivalent terms may be used to mean the same thing – making searching for that concept difficult, requiring multiple ‘Or’ statements.
- They may have multiple meanings (polysemy) especially if “drawn from”, or “applied to”, a wide range of scientific disciplines.
- The different aspects of the resource covered by keywords, i.e., the types or dimensions of keyword applied, may be inconsistent and / or incomplete.

The categorisation system, on the other hand, guarantees that all 7 validated dimensions required are used in the tagging process and that the tags selected are standardised and defined. The toolbox categories also aid browsing of results by enabling sequential filtering using the categories and tags.

In addition, there is a useful link between developing community approved categories for metadata, in this case for characterising resources dealing with sensitive data, and community understood (but implicit) ontologies used in the same area. Categories and ontologies can complement each other—without a common underlying ontology, metadata terms can be interpreted inconsistently, and without defining metadata categories, ontologies may remain implicit and inconsistent. We found, for example, that discussions on the best categorisation to use for scientific disciplines, or data types, exposed the implicit (and different) ontologies being used by different people and is based on the personal views of those in the group. Those would have been obviously rooted in and / or influenced by the language and working assumptions of their discipline(s), and their roles and experiences, (current and previous). That will be more and more the case with interdisciplinary research development and development in research careers*.* Developing categories in metadata can therefore play an important role in describing, understanding and, ultimately, harmonising the implicit ontologies scientists use in thinking about the area of sensitive data.

In the development of the categorisation system, existing ontologies, classifications, and terminologies were taken into consideration (Table 2). However, many more have relationships to the categorisation system. An example is the Subject Resource Application Ontology (SRAO), an application ontology describing subject areas/academic disciplines used within FAIRsharing records by curators and the user community^42^. A first crosswalk has demonstrated considerable agreement between the toolbox category “research field” and subsections of SRAO^42^ and EDAM^15^. The toolbox has been registered as a resource (database) at FAIRsharing, a curated, informative, and educational resource on data and metadata standards, inter-related to databases and data policies (https://fairsharing.org/3577). It is planned to create a collection group of resources (standards, databases, policies) in FAIRsharing linked to the toolbox and the underlying categorisation system. This will also cover relationships to ontologies and classifications.

There is a need to explore the applicability of the toolbox to specific domains. One example could be the European Joint Programme on Rare Diseases (EJP RD), where resources are made progressively FAIR at the record level to support innovative basic, translational and clinical research (https://www.ejprarediseases.org/coordinated-access-data-services/fairification-support/). The goal is to identify, refine and expose core standards for dataset interoperability, asset (data, sample, subject) discovery, and responsible data sharing, concentrating on data level rather than resource level information. Knowledge exchange between EJP RD and the toolbox could be of benefit in exploring the complementary of both approaches in adequately characterising resources linked to sensitive data and thus improving data discoverability.

The first pilot study demonstrated major variation in tagging of resources if independent taggers are assessing the same resource (inter-observer variation). The example of BBMRI has shown that this variation can be considerably reduced if adequate training is performed; which in return is resource intense. Thus, to arrive at a valid and reliable tagging process, there is a necessity for adequate training and support to reduce inter-observer variation. Specific training sets and training programs as well as intercalibration tools need to be developed and implemented and approved by the community.

Another option to be explored should be AI—or ML-algorithms to support automatic (or at least semi-automatic) tagging of resources. It is not easy to use AI/ML in this field due to the multilingualism and the misinterpretation of terms. Often there are different meanings between scientific disciplines and a common backbone for the application of AI/ML is difficult to achieve. It is necessary to come to a common understanding between people involved in the assessment of resources related to sensitive data in all life sciences. Nevertheless, the toolbox can become of major importance for research and application of AI/ML techniques in this field. It may serve as a resource for AI/ML to better find resources in the field by serving as a kind of gold standard to compare with**.** Another promising approach would be to consider a knowledge graph as an intelligent representation. For the categorisation system the approach could be used to interlink categories to a resource (e.g., “source related to sensitive data” has “geographical scope”) and to link individual tags between categories if possible (e.g., “clinical research data” result from “clinical research”). This would give a richer representation of the knowledge behind the categorisation system and the option to be integrated in existing approaches (e.g., OpenAIRE, https://www.openaire.eu/). Therefore, we will consider knowledge graphs as an intelligent knowledge representation of the categorisation system in the future.

A major challenge will be the transition of the toolbox demonstrator to a mature toolbox and ultimately its maintenance, extension, and sustainability. Development of the toolbox demonstrator has been financed by EOSC-Life, but this project will end in 2023. Discussion on sustainability has been initiated with several life-science infrastructures (e.g., BBMRI, EATRIS, ECRIN and ELIXIR, another European Life-Science Infrastructure). Key aspects of sustainability that need to be considered are maintenance of the toolbox portal and tagging tool and of the toolbox content including expert time for tagging as well as human resources to maintain the system. Different approaches are under evaluation: an organization considering the resource core to its operations and taking full responsibility, or a joint ownership across multiple organisations (e.g., multiple RIs) or a community taking responsibility, either funded by future grants or through in-kind contributions from motivated research parties/individuals. Further costs to be covered will include system maintenance, input from a toolbox manager, tagging of resources by experts, as well as advertisement to the envisioned user groups, hardware costs and costs for debugging and major extension of functionality if needed.

## Conclusions

To come to a stable and generally applicable categorisation system and toolbox demonstrator, an iterative process was necessary across life sciences RIs. Approval process started with nominated senior experts from 6 life-science RIs but needs community approval at a larger scale. As the categorisation system is specifying essential metadata for resources about sensitive data, it could be relevant to the FAIR Digital Objects Forum and to the Research Data Alliance (RDA) Metadata Standards Catalog (MSC) Working Group. Initial evaluation of the toolbox demonstrator has been performed with 110 resources from the LS but extension to more resources is needed. Important next steps prior to any realisation of the toolbox will be a broad evaluation of the user acceptance, usability and user-friendliness of the toolbox demonstrator, exploration of AI- or ML- algorithms to support (semi-) automatic tagging of resources and a long-term vision for maintenance and sustainability.

## Supplementary Information

Supplementary Information.

## Data Availability

The detailed results of pilot study 1 are available at Data Intelligence's data repository at the Science Data Bank, http://www.doi.org/10.11922/sciencedb.01529, under an Attribution 4.0 International (CC BY 4.0). Detailed results of pilot study 2 and 3 are included in the Supplementary Information (S3 Report on pilot study 2, S4 Report on pilot study 3).
