# Supplementary material for: An iterative and interdisciplinary categorisation process towards FAIRer digital resources for sensitive life-sciences data
Source: Sci Rep. 2022 Dec 5;12:20989. doi: 10.1038/s41598-022-25278-z (PMC9723099; doi:10.1038/s41598-022-25278-z)
Supplement: Supplementary file 1 — Supplementary Information. [file 41598_2022_25278_MOESM1_ESM.pdf]

Supplementary material for

An iterative and interdisciplinary categorisation process towards FAIRer digital resources for sensitive life-sciences data

Manuscript for Nature Scientific Reports, revised, 12 October 2022

Authors:

Romain David
European Research Infrastructure on Highly Pathogenic Agents (ERINHA), Brussels 1050, Belgium
Christian Ohmann*
European Clinical Research Infrastructure Network (ECRIN), Paris 75013, France
Jan-Willem Boiten
European Advanced Translational Research Infrastructure (EATRIS) / Lygature, Utrecht 3521 AL, The Netherlands
Mónica Cano Abadía
Biobanking and Biomolecular Resources Research Infrastructure (BBMRI-ECRIC), Graz 8010, Austria
Florence Bietrix
European Infrastructure for Translational Medicine (EATRIS), Amsterdam 1081 HZ, The Netherlands
Steve Canham
European Clinical Research Infrastructure Network (ECRIN), Paris 75013, France
Maria Luisa Chiusano
European Marine Biological Resource Centre (EMBRC)
University Federico II of Naples and Stazione Zoologica Anton Dohrn, Naples 80138, Italy
Walter Dastrù
Department of Molecular Biotechnology and Health Sciences, Molecular Imaging Center, University of Torino, Torino I-10125, Italy
Arnaud Laroquette
European Marine Biological Resource Centre (EMBRC), Paris 75252, France
Dario Longo
European Research Infrastructure for Biological and Biomedical Imaging (Euro-Bioimaging) - Institute of Biostructures and Bioimaging. National Research Council of Italy (CNR),Torino, 10126, Italy
Michaela Th. Mayrhofer
Biobanking and Biomolecular Resources Research Infrastructure (BBMRI-ERIC), Graz 8010, Austria
Maria Panagiotopoulou
European Clinical Research Infrastructure Network (ECRIN), Paris 75013, France
Audrey S. Richard
European Research Infrastructure on Highly Pathogenic Agents (ERINHA), Brussels 1000, Belgium
Sergey Goryanin
European Clinical Research Infrastructure Network (ECRIN), Paris 75013, France
Pablo Emilio Verde
Coordination Centre for Clinical Trials, Heinrich Heine University Düsseldorf, Nordrhein-Westfalen 40225, Germany

***Corresponding author:**
Prof. Dr. Christian Ohmann, European Clinical Research Infrastructures Network (ECRIN), Kaiserswerther Strasse 70, 40477, Düsseldorf, Germany, email: christianohmann@outlook.de

Supplementary material

S1: FAIR Guiding principles

(taken from Wilkinson, M., Dumontier, M., Aalbersberg, I. *et al.* The FAIR Guiding Principles for scientific data management and stewardship. *Sci Data* **3,** 160018 (2016). https://doi.org/10.1038/sdata.2016.18)

To be Findable:

F1. (meta)data are assigned a globally unique and persistent identifier
F2. data are described with rich metadata (defined by R1 below)
F3. metadata clearly and explicitly include the identifier of the data it describes
F4. (meta)data are registered or indexed in a searchable resource

To be Accessible:

A1. (meta)data are retrievable by their identifier using a standardized communications protocol
A1.1 the protocol is open, free, and universally implementable
A1.2 the protocol allows for an authentication and authorization procedure, where necessary
A2. metadata are accessible, even when the data are no longer available

To be Interoperable:

I1. (meta)data use a formal, accessible, shared, and broadly applicable language for knowledge representation.
I2. (meta)data use vocabularies that follow FAIR principles
I3. (meta)data include qualified references to other (meta)data

To be Reusable:

R1. meta(data) are richly described with a plurality of accurate and relevant attributes
R1.1. (meta)data are released with a clear and accessible data usage license
R1.2. (meta)data are associated with detailed provenance
R1.3. (meta)data meet domain-relevant community standards

S2: Categories and pre-listed tags for the three different versions of the categorisation system

Category	Version 1<br>(30 November 2020)<br><br>https://doi.org/10.5281/zenodo.4311094	Version 2<br>(12 April 2021)<br><br>https://doi.org/10.5281/zenodo.5506762	Version 3<br>(26 October 2021)<br><br>https://doi.org/10.5281/zenodo.5507324
**Sensitive data type**	Not included	Not included	Personal data
			Environmental data
			Proprietary data
			DURC data
			Classified information
			Other sensitive data
**Resource type**	Legislation & regulations	Legislation and regulations	Legislation and regulations
	Position papers, policies, and principles	Guidelines, recommendations, and policies	Guidelines, recommendations, and policies
	Guidance/recommend-dations		
	Best practice	Descriptions of (best) practice	Descriptions of (best) practice
	Systems, tools & services	Support systems and tools	Support systems and tools
	Background & explanatory material	Other (background) material	Other (background) material
	Repositories/other infrastructures		
	Other resource type		
	Not applicable		
**Research field**	Any		Any research field
	Health research	Clinical research	Clinical research
	Pre-clinical rese4arch	Biomedical research	Biomedical research

		Cell biology, molecular biology, and biochemistry	Cell biology, molecular biology, and biochemistry
	Botanic	Plant and mycological sciences	Plant and mycological sciences
	Zoology	Zoology	Zoology
	Microbiology	Microbiology	Microbiology
		Marine/water biology	Marine/water biology
	Ecology	Ecology and environmental sciences	Ecology
			Environmental sciences
	other	Life science – other topics	Life science – other topics
		Social sciences, legal or ethical research	Social sciences, legal or ethical research
	Not applicable		
**Research design**	Experimental/interventional	Deleted	Deleted
	Observational		
	Secondary research		
	Modelling research		
	Other research designs		
	Not specified/not clear		
	Not applicable		
**Data type**	***Data from/about living humans***	***Data from/about living humans***	***Data from/about living humans***
		Any type of data<br>- sensitive<br>- personal	Any type of data
	Human population-level health or socioeconomic data	Population level health or socioeconomic data	Population level health or socioeconomic data
	Real world or routine health data	Real world or routine health data	Real world or routine health data
	Clinical research data	Clinical research data	Clinical research data
		Public health emergency data	Public health emergency data

	Biobank and registry data	Biobank and sample data	Biobank and sample data
	Data including images	Data with images from humans	Data with images from humans
	Genetic and molecular biology data	Omics related data from living humans	Omics and related data from living humans
		Other specific data type	Other specific data type
		Qualitative research data	
	No data from/about living identifiable human beings	***Data not derived from living humans***	***Data not derived from living humans***
		Other or any data type	Any type of data
			Population level data
			Real world
			Observational/interventional research
			Public health emergency data
			Biobank and sample data
			Data with images
	Omics data generated by basic research	Omics and related data	Omics and related data
		Other or any data type	Other specific data type
	Preclinical research data		
	Organism or species-specific data		
	Ecological/environmental data		
	Other biological data		
	Non-personal sensitive data		
	Other data		
	Other type of data		
	Not specified/not clear		
	Not applicable		

**Stage in data sharing life cycle**	Not applicable	Not applicable	Not applicable
		Whole cycle	Whole cycle
			Primary data registration
	Preparation for data sharing	Preparation and planning for data sharing	Preparation and planning for data sharing
	Planning for data sharing		
	Data preparation at the end of study	Actions at the end of study	Actions at the end of study
	Managing data access	Managing access and data requests for data re-use	Managing access and data requests for data re-use
	Access to data for re-use		
	Transfer of data to a repository		
	Publication of re-use		
	Monitoring data sharing		
	Discovering the data		
	Any		
		Usage, scientific output, and impact of data sharing	
	Other		
**Geographical scope**		Not applicable	Not applicable
	Global	Global	Global
	Continental	Continental	Continental
	Region in the world	Country groupings	Country groupings
	National	National	National
	Sub-national		
	Local		
**Specific topics**			No specific topic
	Data storage agreement	Agreements	Agreements
	Data transfer agreement		

	Data use agreement		
	Attribution and credit for data sharing	Attribution and credit	Attribution and credit
		COVID-19	COVID-19
		Data repositories	Data repositories
	Data Access Committee	Data sharing committees	Data sharing committees
		FAIR and FAIRification	FAIR and FAIRification
	GDPR	GDPR	GDPR
		IP-aspects/licenses	IP-aspects/licenses
		Legal basis for data sharing	Legal basis for data sharing
	Metadata for data sharing	Metadata supporting data sharing	Metadata supporting data sharing
		Privacy protection	Privacy protection
		Research participant involvement	Research participant involvement
	Broad consent		
	Informed consent		
	Alternatives to consent		
	Ethics for data sharing		
	Planning for data re-use		
	Data governance		
	Anonymisation		
	Pseudonymisation		
	De-identificatiuon		
	Repository quality		
	Managing data access		
	Technical & organisational control measures		
	Other topics		
		Dual Use Research of Concern (DURC)	
		ELSI	

		Interventional studies (including clinical trials)	
		Access and Benefit-sharing and the Nagoya protocol	

S3: Report on pilot study 2 (re-tagging exercise)

EOSC-Life WP4

Toolbox demonstrator: Re-tagging exercise of resources linked to sensitive data in the Life-Sciences – a report

Authors: C. Ohmann (ECRIN, retagging coordinator), M. Cano Abadia (BBMRI), F. Bietrix (EATRIS), J.-W. Boiten (EATRIS/Lygature), S. Canham (ECRIN), M.L. Chiusano (EMBRC), W. Dastru (Euro-BioImaging), R. David (ERINHA), A. Laroquette (EMBRC), D. Longo (Euro-BioImaging), M.Th. Mayrhofer (BBMRI), M. Panagiotopoulou (ECRIN), A. Richard (ERINHA), S. Gorianin (ECRIN)

Date: 13 September 2021, update 9 June 2022

Version: Final

1. Introduction

In EOSC-Life WP4 a toolbox demonstrator will be developed, providing links to recommendations, procedures, and best practices, as well as to softwares (tools) dealing with sensitive data in the Life Sciences (1). The objective is to support sensitive data sharing and reuse. It will be based upon a tagging (categorisation) system, allowing consistent labelling and categorisation of resources, in terms most relevant for data sharing tasks and activities. A first version of the categorisation system has been developed (2) and was tested on 110 resources by 12 experts from 6 Life Science Research Infrastructures (3). As a result of the pilot study, major feedback for improvement of the categorisation system has been received and proposals for a revised set of resource tags have been made. A strong need was expressed to reduce observer variation by clearer definitions and better distinction between tags and to keep things as simple as possible. As a consequence, an updated version of the categorisation system has been provided (version 2, (4)). It was decided to re-tag the 110 resources from the pilot study with the updated categorisation system. The re-tagged resources will provide the initial content for the toolbox demonstrator. This report describes the results of the re-tagging exercise.

2. Methods

The re-tagging was performed for the 110 resources from the pilot study by experts from the involved 6 Life Science Research Infrastructures. The involved Research Infrastructures and the coordinating experts as well as the number of resources assessed are listed in table 1:

Research infrastructure	Number of resources assessed	Coordinating experts
BBMRI	25	M.T. Mayrhofer
EATRIS	25	J.-W. Boiten

ECRIN	25	S. Canham
EMBRC	12	M.L. Chiusano
ERINHA	10	R. David
Euro-BioImaging	13	D. Longo

Table 1: Involved Research Infrastructures and coordinating experts

The re-tagging was performed with a tool developed by ECRIN (S. Gorianin). This tool was demonstrated at the EOSC-Life WP4 monthly telephone conference (7.5.2021) and can be accessed via the given link: https://tsdo-admin.ecrin-rms.org/admin/login/?next=/admin/) (credentials are available from ECRIN).

The tool covers three roles, project manager, content managers and taggers. Relevant for the experts doing the re-tagging was only the "tagger's role". For preparation of the re-tagging procedure, metadata of the 110 resources have been uploaded to the toolbox demonstrator (basic bibliographic data). The re-taggers got access only to the resources they had been allocated to. For these resources, the re-taggers were able to perform the re-tagging by selecting pre-specified tags from given lists. When the re-tagging has been completed, the experts are presented with an overview of the tags entered for final confirmation. The information to access the tool (username, initial password) was directly sent to the experts by the project coordinator (Christian Ohmann).

Unlike in the pilot study, in which tagging was performed under experimental conditions with experts blinded to each other within a Research Infrastructure, the re-tagging was executed as an uncontrolled procedure. For each involved Research Infrastructure, one responsible coordinating expert, preferably the main contact person that took part in the pilot study, performed the re-tagging. It was allowed to use material from the pilot study and discussion between experts in a Research Infrastructure was possible and desired. The re-tagging was intended to be as complete and precise as possible, having in mind that **this is providing the initial content for the toolbox demonstrator** with the resources and tags openly available.

In summary:

- Each of the 6 Life Science Research Infrastructures nominated **one expert**, responsible for co-ordinating and performing the re-tagging (preferably the primary contact person involved in the pilot study).
- ECRIN provided the computer tool for re-tagging with the bibliographic metadata of all 110 resources included. Access for re-taggers was restricted to the resources allocated to their Research Infrastructure. Access details were sent to the individual re-taggers by the project coordinator from ECRIN.
- The re-tagging procedure was launched by the project coordinator on **21 May 2021.** The re-tagging of all 110 resources was completed **6 July 2021.**

3. Results

3.1 Tags assigned to the resources

The assigned tags were analysed for the individual categories of the updated categorisation system (version 2).

In table 2, the results for category “resource type” are presented:

**Category 1 “Resource type”**	**Research infrastructure**						
	BBMRI (n=25)	EATRIS (n=25)	ECRIN (n=25)	EMBRC (n=12)	ERINHA (n=10)	Euro-Bio Imaging (n=13)	Total (n=110)
**Tags**							
Legislation and regulations	3	3	0	0	3	2	11
Guidelines, recommendations and policies	3	11	8	4	7	7	40
Description of best practice	2	6	4	3	3	7	25
Support systems and tools	9	6	8	6	3	6	38
Other (background) material	14	1	9	0	3	0	27
Total	30	27	29	13	19	22	141
**No. of tags per resource**							
1	22	23	21	11	3	5	85
2	2	2	4	1	5	7	21
>= 3	1	0	0	0	2	1	4

Table 2: Results of re-tagging of the 110 resources for the category “resource type”

For the majority of resources (77%), only one “resource type” was allocated. Assigning three or more “resource types” to one specific resource occurred very rarely. All tags were used at least 10 times, most frequently “guidelines, recommendations and policies” (36%) and “support systems and tools” (35%).

In table 3, the results for the category “research field” are summarised:

**Category “Research field”**	**Research infrastructure**						
	BBMRI (n=25)	EATRIS (n=25)	ECRIN (n=25)	EMBRC (n=12)	ERINHA (n=10)	Euro-Bio Imaging (n=13)	Total (n=110)
**Tags**							
Clinical research	16	15	25	0	9	13	78
Biomedical research	20	19	13	0	9	1	62
Cell biology, molecular biology and biochemistry	1	4	2	0	9	0	16
Plant and mycological sciences	1	2	2	0	10	0	15
Zoology	1	2	2	0	8	0	13
Microbiology	1	2	2	0	9	0	14
Marine/water biology	1	2	2	0	2	0	7
Ecology and environmental sciences	1	2	2	3	3	0	11
Life science - other topics	15	3	2	9	9	0	38
Social sciences, legal or ethical research	8	9	5	0	4	0	26

Total	65	60	57	12	72	14	280
**No. of tags**							
1	8	10	11	12	0	12	53
2	7	9	10	0	0	1	27
>= 3	10	6	4	0	10	0	30

Table 3: Results of re-tagging of the 110 resources for the category "research field"

For around half of the resources (48%) only one "research field" was allocated. Most frequently "clinical research" (71%) and "biomedical research" (56%) were selected as a "research field". As a consequence of the selection of resources, "marine/water biology" (6%), "ecology and environmental sciences" (10%), "zoology" (12%), "microbiology" (13%), "plant and mycological sciences" (14%) and "cell biology, molecular biology and biochemistry" (15%) were rarely used. Interestingly, for 35% of the resources "life science – other topics" was used.

In table 4, the results for the category "data type" are presented:

**Category "Data type"**	**Research infrastructure**						
	BBMRI (n=25)	EATRIS (n=25)	ECRIN (n=25)	EMBRC (n=12)	ERINHA (n=10)	Euro-Bio Imaging (n=13)	Total (n=110)
Data about living humans							
- Any type of data	11	8	13	0	9	0	41
- Sensitive	0	0	0	0	9	0	9
- Personal	0	6	1	0	0	0	7
- Sensitive & personal	11	2	12	0	0	0	25
- Specific data type	14	17	12	0	1	3	57
- Population level health/or socio-economic data	6	1	0	0	0	0	7
- Real world or routine health data	5	4	3	0	0	1	13
- Clinical research data	11	4	10	0	0	4	29
- Public health emergency data	0	1	1	0	0	1	3
- Biobank and sample data	13	1	2	0	0	1	17
- Data with images of humans	6	3	1	0	0	11	21
- Qualitative research data	0	0	0	0	0	0	0
- Omics and related data (from living humans)	6	8	1	2	0	1	18
- Other specific data type	7	1	0	0	1	0	9

Data not derived from living humans<br>- Omics and related data<br>- Other or any data type (non-human)	0<br><br>0<br><br>0	0<br><br>0<br><br>0	0<br><br>0<br><br>0	12<br><br>2<br><br>12	0<br><br>0<br><br>0	0<br><br>0<br><br>0	12<br><br>2<br><br>12
Total	25	25	25	12	10	13	110
**No. of tags**							
1	4	20	9	10*	10*	7	60
2	14	4	14	2	0	5	39
>= 3	7	1	2	0	0	1	11

Table 4: Results of re-tagging of the 110 resources for the category "data type"
***Changed in this update from 9.6.2022**

For around half of the 110 resources, specific data types related to humans were allocated. Only one tag was applied for 55% of the resources. Again, as a consequence of the selection of resources, data from non-living humans played a minor role (11%). From the specific data types, "qualitative research data" (0%), "public health emergency data" (3%) and "population level health/or socioeconomic data" (6%), were rarely selected.

In table 5, the results for the category "stage in data sharing life cycle" are summarised:

**Category "Stage in data sharing life cycle"**	**Research infrastructure**						
	BBMRI (n=25)	EATRIS (n=25)	ECRIN (n=25)	EMBRC (n=12)	ERINHA (n=10)	Euro-Bio Imaging (n=13)	Total (n=110)
**Tags**							
Not applicable	3	2	16	0	1	0	22
Whole cycle	14	5	0	4	3	4	30
Preparation and planning for data sharing	8	8	3	5	1	3	28
Actions at the end of a study	0	1	6	0	0	1	8
Managing data access and requests for data re-use	0	9	0	3	4	5	21
Usage, scientific output and impact of DS	0	0	0	0	1	0	1
Total	25	25	25	12	10	13	110
**No. of tags**							
1	25	25	25	12	10	13	110
2	0	0	0	0	0	0	0
>= 3	0	0	0	0	0	0	0

Table 5: Results of re-tagging of the 110 resources according to the category "stage in data sharing life cycle"

All resources had precisely one tag assigned in this category (100%), however, for a considerable number of resources (20%), the category was “not applicable”. More often “whole cycle” (27%), “preparation and planning for data sharing” (25%) and “managing data access and requests for data re-use” (19%) were used. “Usage, scientific output and impact of data sharing” was allocated to only one resource (1%).

In table 6, the results for category “geographical scope” are presented:

**Dimension “Geographical scope”**	**Research infrastructure**						
	BBMRI (n=25)	EATRIS (n=25)	ECRIN (n=25)	EMBRC (n=12)	ERINHA (n=10)	Euro-Bio Imaging (n=13)	Total (n=110)
**Tags**							
Not applicable	0	3	11	3	3	0	20
Global	13	10	6	5	4	5	43
Continental<br>- Europe<br>- North-America	12<br>12<br>0	2<br>1<br>0	0<br>0<br>0	1<br>1<br>0	2<br>2<br>0	3<br>2<br>2	20<br>18<br>2
Country grouping<br>- EU<br>- LMIC	0<br>0<br>0	5<br>5<br>0	3<br>2<br>1	3<br>3<br>0	0<br>0<br>0	0<br>0<br>0	11<br>10<br>1
National<br>- US<br>- UK<br>- Sweden<br>- Netherlands<br>- Germany<br>- France	0<br>0<br>0<br>0<br>0<br>0<br>0	5<br>1<br>0<br>0<br>4<br>0<br>0	5<br>2<br>2<br>0<br>0<br>1<br>0	0<br>0<br>0<br>0<br>0<br>0<br>0	1<br>1<br>0<br>0<br>0<br>0<br>0	5<br>2<br>0<br>1<br>0<br>1<br>1	16<br>6<br>2<br>1<br>4<br>2<br>1
**No. of tags**							
1	25	25	25	12	10	13	110
2	0	0	0	0	0	0	0
>= 3	0	0	0	0	0	0	0

Table 6: Results of re-tagging of the 110 resources for the category “geographical scope”

Only one tag was assigned to all resources (100%). In a considerable portion of resources this category was “not applicable” (18%). Most often the tag “global” (39%) was used.

In table 7, the results for the category “specific topics” are summarised:

**Category “Specific topics”**	**Research infrastructure**						
	BBMRI (n=25)	EATRIS (n=25)	ECRIN (n=25)	EMBRC (n=12)	ERINHA (n=10)	Euro-Bio Imaging (n=13)	Total (n=110)
**Tags**							
Agreements	5	1	0	2	0	1	9
Attribution and credit	0	0	2	0	0	1	3
COVID-19	3	2	1	0	1	0	7

Data repositories	2	6	3	7	4	8	30
Data sharing committees	1	6	0	1	6	1	15
Dual use Research of concern (DURC)	0	1	0	0	9	0	10
ELSI (Ethical, legal and social aspects)	24	14	6	3	5	1	53
FAIR and FAIRification	1	1	0	0	3	9	14
GDPR	7	9	0	0	0	1	17
IP-aspects/licences	1	0	0	0	2	0	3
Legal basis for data sharing	6	2	3	0	4	2	17
Interventional studies (including clinical trials)	0	0	7	0	0	0	7
Metadata supporting data sharing	0	4	0	3	2	5	14
Access and benefit-sharing and the Nagoya protocol	2	0	0	5	0	2	9
Privacy protection	1	8	11	1	2	2	25
Research participant involvement	5	0	1	0	3	0	9
Total	58	54	34	22	41	33	242
**No. of tags**							
1	13	6	18	5	2	1	45
2	4	10	6	4	0	8	32
>= 3	8	9	1	3	8	4	33

Table 7: Results of re-tagging of the 110 resources for the category "specific topics"

For the majority of resources (59%), two or more tags were assigned from this category. Most frequently, "ELSI" (48%) was used, followed by "data repositories" (27%) and "privacy protection" (23%). Rarely applied were "IP-aspects/licenses" (3%), "attributes and credit" (3%), "COVID-19" (6%), "interventional studies" (6%), "agreements" (8%), "access and benefit sharing and the Nagoya protocol" (8%) and "research participant involvement" (8%).

3.2 Feedback by the experts

Specific feedback was received from five Research Infrastructures. The comments are included in the appendix.

Most frequently, feedback was related to the content of and structure of the categorisation system.

- Category "data type"

This category was seen as too complex and created major discussion. Several experts strongly suggested to better define this category and to make it easier applicable. The selection between "personal" and "sensitive" only for "any type of data" and not for the other data types was seen as critical. It was not clear where to allocate genomic resources. For "sensitive data", the definition from David et al. (https://zenodo.org/record/3922069#.YCJU7ehKg2w) was proposed and it was suggested to add an extra category "type of sensitive data" with a list of the main 4-6 types of sensitive data (e.g. sensitive personal data, environmental data, business data, DURC related data, sensitive classified information). This would make the categorisation a bit more complex but easier to apply. In addition, it was suggested to include the tag "any type of data".

- Category “research field”

One expert had hard times to allocate “research field” in general. Many resources cover all of health research and are difficult to tag. One suggestion was to include the tag “any research field”. Another expert raised the point that “research field” and “data type” appear to have some overlap.

- Category “data sharing life cycle”

For one expert, the stage in the data sharing life cycle was difficult to select because many resources did not fall into one category. It was argued that some of the tags are not defined precisely enough.

- Category “geographical scope”

It was not clear to one expert what determines the geographical scope, the origin of the resource or the applicability of the resource. It was also argued that the difference between “not applicable” and “global” is vague.

- Category “specific topics”

One expert saw “ELSI” as too generic, covering nearly everything. “Interventional studies” were seen more as a “data type” not to be included under “specific topics”. It was also criticised that it was mandatory to select at least one specific point from the list, even if not applicable.

The user interface and the functionality of the tagging tool was generally considered as positive by the experts. Some suggestions were made to reduce clicks if not needed. A few requests for improving functionality were made, which will be included in the requirements for an update of the tagging tool.

One expert raised a major point concerning the procedure of re-tagging. It was argued that the re-tagging should be done by one trained person or by a permanent small board, indicating however, that this may be too resource intensive.

Finally, some comments related to the selections of resources were received. It was suggested not to include resources which are behind paywalls.

4. Discussion

As a consequence of the re-tagging exercise and the feedback received, the decision to revise the categorisation system and to provide an updated version 3 was taken. A major adaptation was suggested for the category “data type”, which was seen as not adequately structured by some of the experts. To simplify and to clarify this, a new category “sensitive data type” will be introduced, listing the main types of sensitive data. With this new category, the original category “data type” could be simplified and the number of tags in “specific topics” could be reduced. In addition, the other comments related to the different categories will be taken into consideration, resulting in an updated version of the categorisation system, to be added in short form in table 8:

Table 8: Short form of the updated categorisation system (version 3)
(see: https://doi.org/10.5281/zenodo.5507324)

The re-tagged 110 resources constitute the basic content for the toolbox demonstrator. This demonstrator is currently developed by ECRIN according to the defined requirements (5) and based upon existing software tools, such as the metadata repository developed by ECRIN (http://51.210.99.18/). Next steps will be the development of a sustainability plan involving interested Research Infrastructures (first meetings have taken place) and a user satisfaction/user feedback survey with broad involvement of the Life Science community represented in EOSC-Life.

5. References

1. J.-W. Boiten, C. Ohmann, A. Adeniran, S. Canham, M. Cano Abadia, G. Chassang, M.L. Chiusano, R. David, M. Fratelli, P. Gribbon, P. Holub, R. Ludwig, M. Th. Mayrhofer, M. Matei, A. Merchant, M. Panagiotopoulou, L. Pireddu, A. Sanchez Pla, I. Schlünder, T. George, H. Wagener (2021, January 31). EOSC-LIFE WP4 TOOLBOX: Toolbox for sharing of sensitive data - a concept description. Zenodo. http://doi.org/10.5281/zenodo.4483694

2. C. Ohmann, S. Canham, J.-W. Boiten, M. Cano Abadía, G. Chassang, M.L. Chiusano, R. David, M. Th. Mayrhofer, L. Pireddu (2020, December 8). EOSC-Life WP4 Toolbox: Categorisation system for resources to be referenced in the toolbox for sharing of sensitive data (Version 1). Zenodo. http://doi.org/10.5281/zenodo.4311094

3. C. Ohmann (ECRIN, pilot study coordinator), M. Cano Abadia (BBMRI), F. Bietrix (EATRIS), J.-W. Boiten (EATRIS/Lygature), S. Canham (ECRIN), M.L. Chiusano (EMBRC), W. Dastru (Euro-BioImaging), R. David (ERINHA), A. Laroquette (EMBRC), D. Longo (Euro-BioImaging), M.Th. Mayrhofer (BBMRI), M. Panagiotopoulou (ECRIN), A. Richard (ERINHA), P. Verde (University Düsseldorf, statistical support) (2021). EOSC-Life WP4 Toolbox: Pilot study for the evaluation of a tagging system characterising resources linked to sensitive data in the life-sciences. https://docs.google.com/document/d/1DbzkjmsSy7Wvrlchs6C__VpBjMUGa4GL/edit

4. C. Ohmann (ECRIN, pilot study coordinator), M. Cano Abadia (BBMRI), F. Bietrix (EATRIS), J.-W. Boiten (EATRIS/Lygature), S. Canham (ECRIN), M.L. Chiusano (EMBRC), W. Dastru (Euro-BioImaging), R. David (ERINHA), A. Laroquette (EMBRC), D. Longo (Euro-BioImaging), M.Th. Mayrhofer (BBMRI), M. Panagiotopoulou (ECRIN), A. Richard (ERINHA) (2021). The EOSC-Life WP4 toolbox: Update of the categorisation system. https://docs.google.com/document/d/13cVXp2Dyntpyg1r3md7-YDllTEI0mKfX/edit

5. S. Gorianin (ECRIN) (coordinator), M. Cano Abadia (BBMRI), S. Canham (ECRIN), M.L. Chiusano (EMBRC), R. David (ERINHA), M. Th. Mayrhofer (BBMRI), H. Munneke (Lygature/EATRIS), C. Ohmann (ECRIN), M. Panagiotopoulou (ECRIN), H. Wagener (Charité) (2021, April 9, version 0.2). EOSC-Life WP4 Toolbox Technical Subcommittee: Recommendations for technical implementation. https://docs.google.com/document/d/10s7IuOlwMEJGWCg7QGUmnqOTi2HagyA2/edit

S4: Report on pilot study 3 (re-re-tagging exercise)

EOSC-Life WP4

Toolbox demonstrator: Re-re-tagging exercise of resources linked to sensitive data in the Life-Sciences – a report

Authors: C. Ohmann (ECRIN, retagging coordinator), M. Cano Abadia (BBMRI), F. Bietrix (EATRIS), J.-W. Boiten (EATRIS/Lygature), S. Canham (ECRIN), M.L. Chiusano (EMBRC), W. Dastru (Euro-BioImaging), R. David (ERINHA), A. Laroquette (EMBRC), D. Longo (Euro-BioImaging), M.Th. Mayrhofer (BBMRI), M. Panagiotopoulou (ECRIN), A. Richard (ERINHA), S. Gorianin (ECRIN)

Date: 31 January 2022

Version: Final

1. Introduction

In EOSC-Life WP4 a toolbox demonstrator is being developed, providing links to recommendations, procedures, and best practices, as well as to software (tools) dealing with sensitive data in the Life Sciences (1). The objective is to support sensitive data sharing and reuse. It is based upon a tagging (categorisation) system, allowing consistent labelling and categorisation of resources, in terms most relevant to data sharing tasks and activities. A first version of the categorisation system has been developed (2) and was tested on 110 resources by 12 experts from 6 Life Science Research Infrastructures (3). As a result of the pilot study, major feedback for improvement of the categorisation system was received and proposals for a revised set of resource tags were made. As a consequence, an updated version of the categorisation system has been provided (version 2, (4)). It was decided to re-tag the 110 resources from the pilot study with the updated categorisation system (5). Again, major feedback for improvements to the categorisation system was received from this second pilot study leading to a new version of the categorisation system (version 3) (6). Finally, the 110 resources were re-re-tagged with the categorisation system version 3. The process is summarised in table 1:

**Step 1**	**Date**	Reference
Categorisation system 1	30 November 2020	http://doi.org/10.5281/zenodo.4483694
Concept for toolbox	31 January 2021	http://doi.org/10.5281/zenodo.4311094

Pilot study protocol for evaluation of categorisation system 1	8 December 2020	https://zenodo.org/record/4311114#.YH011-gzaUk
Pilot study 1 (tagging) for evaluation of categorisation system 1	3 March 2021	Internal report
Categorisation system 2	12 April 2021	https://zenodo.org/record/5506762#.YUCy7p0zaUl
Pilot study 1 report submitted to Data Intelligence Journal	6 July 2021	Under evaluation
Pilot study 2 (re-tagging) for evaluation of categorisation system 2	13 September 2021	Internal report
Categorisation system 3	26 October 2021	https://zenodo.org/record/5507324#.YYTwg2DMKUk
Pilot study 3 (re-re-tagging) for evaluation of categorisation system 3	10 December 2021	Internal report

Table 1: Stepwise procedure for development of the toolbox

This report describes the results of the re-re-tagging exercise, using version 3 of the categorisation system.

2. Methods

The re-re-tagging was performed for the 110 resources from the pilot study by experts from the involved 6 Life Science Research Infrastructures. The involved Research Infrastructures and experts as well as the number of resources assessed are listed in table 2:

Research infrastructure	Number of resources to be assessed	Experts
BBMRI	25	M.T. Mayrhofer
EATRIS	25	J.-W. Boiten
ECRIN	25	S. Canham
EMBRC	12	M.L. Chiusano
ERINHA	10	R. David
Euro-BioImaging	13	D. Longo

Table 2: Involved Research Infrastructures and coordinating experts

The re-re-tagging was performed with a computer tool developed by ECRIN (S. Gorianin). Access for re-re-taggers was restricted to the resources allocated to their Research Infrastructure. This tool can be accessed via the given link (https://tsdo-admin.ecrin-rms.org/admin).

The start signal for the re-re-tagging procedure was given on **2 November 2021**. The re-tagging of the resources was completed **30 November 2021.**

The results of the re-re-tagging procedure will constitute the initial content for the toolbox demonstrator and the tagging of these resources will become openly available.

3. Results

One resource was not tagged, so in total 109 resources have been available for the analysis.

3.1 Tags assigned to the resources

The number of assigned tags is analysed for the individual categories of the categorisation system (version 3).

In Table 3, the results for category "sensitive data type" are presented:

**Category "Sensitive data type"**	**Research infrastructure**						
	BBMRI (n=25)	EATRIS (n=25)	ECRIN (n=25)	EMBRC (n=12)	ERINHA (n=10)	Euro-Bio Imaging (n=13)	Total (n=110)
**Tags**							
Personal data	24	24	25	0	2	12	87
Environmental data	0	2	2	1	2	0	7
Proprietary data	0	2	1	0	!	0	4
DURC data	0	3	1	0	9	0	13
Classified information	0	2	1	0	2	0	5
Other sensitive data	23	2	1	12	1	1	40
Total	47	35	31	12	17	12	156
**No. of tags**							
1	1	23	23	11	7	13	78
2	23	0	1	1	2	0	27
>= 3	0	2	1	0	1	0	4

Table 3: Results of re-re-retagging of the 110 resources for the category "sensitive data type"

For the majority of resources (72%), only one "sensitive data type" was allocated. Assigning three or more "sensitive data types" to one specific resource occurred very rarely (4%). 80% of the resources were characterised as dealing with "personal data". For more than 1/3 of the resources (37%) "other sensitive data" was selected, indicating that the prespecified list of tags needs to be extended to allow better coverage. From the rest of the prespecified tags ("environmental data", "proprietary data", "DURC data", "classified information") only "DURC data" was used in more than 10% (12).

Table 4 summarises the results for the category “resource type”.

Category “Resource type”	Research infrastructure						
	BBMRI (n=25)	EATRIS (n=25)	ECRIN (n=25)	EMBRC (n=12)	ERINHA (n=10)	Euro-Bio Imaging (n=13)	Total (n=110)
**Tags**							
Legislations & regulations	4	4	0	0	4	2	14
Guidelines, recommendations and policies	6	14	8	11	6	7	52
Description of (best) practice	5	3	4	1	4	6	23
Support systems & tools	8	5	8	2	1	6	30
Other (background) material	18	0	6	0	3	1	28
Total	41	26	26	14	18	22	147
**No. of tags**							
1	14	24	24	10	3	5	80
2	7	1	1	2	6	7	24
>= 3	3	0	0	0	1	1	5

Table 4: Results of re-re-tagging of the 110 resources for the category “resource type”

For the majority of resources (73%), only one “resource type” was allocated. Assigning three or more “resource types” to one specific resource occurred very rarely (5%). All tags were used at least 10 times, most frequently “guidelines, recommendations and policies” (48%), followed by “support systems and tools” (28%), “other (background) material (26%) and description of (best) practice (23%).

In Table 5, the results for the category “research field” are summarised:

Category “Research field”	Research infrastructure						
	BBMRI (n=25)	EATRIS (n=25)	ECRIN (n=25)	EMBRC (n=12)	ERINHA (n=10)	Euro-Bio Imaging (n=13)	Total (n=110)
**Tags**							
Any research field	4	5	5	0	6	0	20
Clinical research	11	10	20	0	0	13	54
Biomedical research	14	17	12	0	1	1	45
Cell biology, molecular biology and biochemistry	0	7	0	0	2	0	9
Plant and mycological sciences	0	1	0	4	1	0	6
Zoology	0	0	0	4	1	0	5
Microbiology	0	1	0	4	2	0	7
Marine/water biology	0	1	0	11	1	0	13
Ecology	0	1	0	4	1	0	6

Environmental sciences	0	0	0	1	0	0	1
Life science - other topics	15	1	0	0	1	0	17
Social sciences, legal or ethical research	7	5	4	0	2	0	18
Total	51	49	41	28	18	14	201
**No. of tags**							
1	8	8	12	8	6	12	54
2	9	12	10	0	1	1	33
>= 3	7	5	9	4	3	0	28

Table 5: Results of re-re-tagging of the 110 resources for the category "research field"

For half of the resources (50%) only one "research field" was allocated. In 30% of the cases two tags and in 26% three or more tags were used. Most frequently "clinical research" (50%) and "biomedical research" (41%) were selected as "research field". Apart from "social sciences, legal or ethical research" (17%) and "marine/water biology" (12%), all other "research fields" were used less than 10 times. Only one resources was related to "environmental sciences". In 16% of the resources no tag from the prespecified list could be applied and "life science – other topics" was allocated. The under-use of other research fields apart from "clinical research" and "biomedical research" is due to the selection process of the resources, which was dominated by the medical RIs.

In Table 6, the results for the category "data type" are presented:

**Category "Data type"**	**Research infrastructure**						
	BBMRI (n=25)	EATRIS (n=25)	ECRIN (n=25)	EMBRC (n=12)	ERINHA (n=10)	Euro-Bio Im.(n=13)	Total (n=110)
Data about or from living humans	24	24	25	0	3	13	89
• Any type of data	20	9	9	0	3	0	41
• Population level health/or socio-economic data	0	3	0	0	0	0	3
• Real world or routine health data	0	5	9	0	0	1	15
• Clinical research data	2	4	14	0	0	6	26
• Observational/inter-ventional research	0	0	0	0	0	0	0
• Public health emergency data	0	2	0	0	0	0	2
• Biobank and sample data	4	1	5	0	0	1	11
• Data with images	1	2	5	0	0	11	19
• Omics and related data	1	6	0	0	0	1	8
• Other specific data type	1	0	0	0	0	0	1

Total	29	32	42	0	3	20	126
Data not derived from living humans	0	1	0	12	7	0	20
• Any type of data	0	1	0	9	6	0	16
• Population level health/or socio-economic data	0	0	0	1	0	0	1
• Real world or routine health data	0	0	0	0	2	0	2
• Clinical research data	0	0	0	0	0	0	0
• Observational/inter-ventional research	0	0	0	6	1	0	7
• Public health emergency data	0	0	0	0	2	1	3
• Biobank and sample data	0	0	0	0	2	0	2
• Data with images	0	0	0	0	0	0	0
• Omics and related data	0	0	0	2	1	0	3
• Other specific data type	0	0	0	1	1	0	2
Total	0	1	0	19	15	1	36
**No. of tags**							
1	22	20	17	7	6	7	79
2	0	3	0	3	2	5	13
>= 3	2	2	8	2	2	1	17

Table 6: Results of re-re-tagging of the 110 resources for the category "data type"

Only one "data type" was selected for 72% of the resources. The overwhelming majority delt with data about or from living humans (82%), only 18% with data not derived from living humans. For resources linked to data about or from living humans (n=89), "any to type of data" was selected most often (46%), followed by "clinical research data (29%), "data with images" (21%) and "real world or routine health data" (17%). If resources were related to data not derived from living humans (n=20), "any type of data" was indicated most frequently (80%), followed by "observational/interventional research" (35%).

In Table 7, the results for the category "stage in data sharing life cycle" are summarised:

**Category "Stage in data sharing life cycle"**	**Research infrastructure**						
	BBMRI (n=25)	EATRIS (n=25)	ECRIN (n=25)	EMBRC (n=12)	ERINHA (n=10)	Euro-Bio Imaging (n=13)	Total (n=110)
**Tags**							

Not applicable	5	13	19	0	3	0	40
Whole cycle	12	1	0	4	1	4	22
Primary data registration	0	3	0	0	0	0	3
Preparation and planning for data sharing	7	5	6	8	3	3	32
Actions at the end of a study	0	3	0	0	0	1	4
Managing data access and requests for data re-use	0	0	0	0	3	5	8
Total	24	25	25	12	10	13	109
**No. of tags**							
1	24	25	25	12	10	13	109
2	0	0	0	0	0	0	0
>= 3	0	0	0	0	0	0	0

Table 7: Results of re-re-tagging of the 110 resources according to the category "stage in data sharing life cycle"

Exactly one tag was allocated to all resources (100%), however, for a considerable number of resources, the tag selected was "not applicable" (37%). Most often "preparation and planning for data sharing" (29%) and "whole cycle" (20%) were selected. Because this category seems not to be very discriminative, it needs to be reconsidered or may need to be defined a bit deeper.

In Table 8, the results for category "geographical scope" are presented:

**Dimension "Geographical scope"**	**Research infrastructure**						
	BBMRI (n=25)	EATRIS (n=25)	ECRIN (n=25)	EMBRC (n=12)	ERINHA (n=10)	Euro-Bio Imaging (n=13)	Total (n=110)
**Tags**							
Not applicable	1	0	12	0	3	0	16
Global	13	10	5	9	5	4	46
Continental	10	0	2	3	1	4*	20
- Europe	10	0	2	3	1	2	18
- North-America	0	0	0	0	0	3	3
Country grouping	0	7	0	0	0	0	7
- EU	0	7	0	0	0	0	7
- LMIC	0	0	0	0	0	0	0
National	0	8	6	0	1	5	20
- US	0	3	1	0	1	2	7
- UK	0	0	3	0	0	0	3
- Sweden	0	0	0	0	0	1	1
- Netherlands	0	5	0	0	0	0	5
- Germany	0	0	1	0	0	1	2
- France	0	0	0	0	0	1	1
- Austria	0	0	1	0	0	0	1
Total	24	25	25	12	10	13	109

**No. of tags**							
1	24	25	25	12	10	12	108
2	0	0	0	0	0	1	1
>= 3	0	0	0	0	0	0	0

Table 8: **Results of re-re-tagging of the 110 resources for the category "geographical scope"**
*one resource with 2 continents

For all but one resource only one tag was assigned. Most often, "global" was assigned (42%). "Continental" and "national" were considered equally often (18%), followed by "country grouping (6%).

In Table 9, the results for the category "specific topics" are summarised:

**Category "Specific topics"**	**Research infrastructure**						
	BBMRI (n=25)	EATRIS (n=25)	ECRIN (n=25)	EMBRC (n=12)	ERINHA (n=10)	Euro-Bio Imaging (n=13)	Total (n=110)
**Tags**							
No specific topic	6	1	7	0	4	0	18
Agreements	7	1	0	8	2	1	19
Attribution and credit	0	1	2	0	1	1	5
COVID-19	3	0	0	0	1	0	4
Data repositories	4	6	5	3	2	9	29
Data sharing committees	3	8	0	0	3	1	15
FAIR and FAIRification	2	1	0	0	1	7	11
GDPR	6	7	0	0	2	2	17
IP-aspects/licences	0	0	0	0	1	0	1
Legal basis for data sharing	5	5	2	7	5	2	26
Metadata supporting data sharing	0	4	0	0	3	5	12
Privacy protection	9	9	11	0	2	2	33
Research participant involvement	9	1	1	0	1	0	12
Total	54	44	28	18	28	30	202
**No. of tags**							
1	16	9	22	6	5	3	61
2	3	13	3	6	0	6	31
>= 3	5	3	0	0	5	4	17

Table 9: **Results of re-re-tagging of the 110 resources for the category "specific topics"**

For the majority of resources (56%), only one tag was assigned from the prespecified list. In 28% of the cases two tags and in 16% three or more tags were used. Apart from "IP-aspects/licenses", "COVID-19" and "attribution and credit", all other tags were used at least 10 times, most often "privacy protection" (30%), followed by "data repositories" (27%) and "legal basis for data sharing" (24%).

3.2 Feedback by the experts

Additional written feedback was received from four Research Infrastructures.

In summary, the application of the computer tagging tool was user-friendly and efficient. Several improvements for the tool were suggested:

a) The status of the tagging should be made more visible
b) It should be possible to select both human and non-human data
c) For some categories too much clicking is needed (e.g., geographical scope)
d) There are some duplicates in the tag lists, which needs to be corrected
e) The buttons at the bottom of the screen are misleading and have to be made clearer (e.g., “save and approve”)
f) The messaging function should be checked if it works correct
g) Some URLs were not properly working

It is planned to improve the tool according to these suggestions in the next version.

The categorisation system version 3 raised no major concerns, indicating that after 3 cycles of improvement the system converged and can now be considered as stable. The following minor points were brought up:

a) Add additional tags for “specific topics (e.g., “general frameworks/principles for data sharing”, “ethical frameworks/principles”)
b) Differentiate more clearly between “global” and “not applicable” for “geographical scope”
c) Include “any data” as extra tag to “data type”.
d) It should be possible to select “human” and “non-human data” in “data type”
e) The category “stage in data sharing life cycle” is still difficult to apply. For some of the experts the tags were difficult to select. This has to better explained in the documentation.
f) Multiple tags should be selectable for “stage in data sharing life cycle”
g) The tags for “sensitive data type” need some extension. It was suggested to add “biological data” and “guidelines, rules” to “sensitive data type”, even if there is some overlap.
h) For the category “specific topics” the tag “no specific topics” should be renamed in “no specific topics from the list below”

The points raised above will be taken into consideration for the next version of the categorisation system.

4. Conclusions

The re-re-tagging exercise has demonstrated good maturity and convergence of the categorisation system as well as the ease of use of the tagging system. Minor proposals for improvement have been made and will be taken into consideration with the next version.

The re-re-tagged 110 resources will constitute the basic content for the toolbox demonstrator. This demonstrator has been developed by ECRIN according to defined requirements and based upon existing software tools, such as the metadata repository developed by ECRIN (https://crmdr.org/). Next steps will be the development of a sustainability plan involving interested Research Infrastructures (a first meeting has taken place) and a user satisfaction/usability survey with broad involvement of the Life Science community represented in EOSC-Life. An update of the categorisation system (version 4) as well as the update and extension of the toolbox demonstrator will be taken into consideration when considerable progress has been made with respect to the sustainability plan.

5. References

1. J.-W. Boiten, C. Ohmann, A. Adeniran, S. Canham, M. Cano Abadia, G. Chassang, M.L. Chiusano, R. David, M. Fratelli, P. Gribbon, P. Holub, R. Ludwig, M. Th. Mayrhofer, M. Matei, A. Merchant, M. Panagiotopoulou, L. Pireddu, A. Sanchez Pla, I. Schlünder, T. George, H. Wagener (2021, January 31). EOSC-LIFE WP4 TOOLBOX: Toolbox for sharing of sensitive data - a concept description. Zenodo. http://doi.org/10.5281/zenodo.4483694

2. C. Ohmann, S. Canham, J.-W. Boiten, M. Cano Abadía, G. Chassang, M.L. Chiusano, R. David, M. Th. Mayrhofer, L. Pireddu (2020, December 8). EOSC-Life WP4 Toolbox: Categorisation system for resources to be referenced in the toolbox for sharing of sensitive data (Version 1). Zenodo. http://doi.org/10.5281/zenodo.4311094

3. C. Ohmann, R. David, M. Cano Abadia, F. Bietrix, J.-W. Boiten, S. Canham, M.L. Chiusano, W. Dastru, A. Laroquette, D. Longo, M.Th. Mayrhofer, M. Panagiotopoulou, A. Richard, P. Verde (2021). Pilot study on the intercalibration of a categorisation system for FAIRer digital objects related to sensitive data in the life sciences", Data Intelligence Journal, under review.

4. C. Ohmann (ECRIN, pilot study coordinator), M. Cano Abadia (BBMRI), F. Bietrix (EATRIS), J.-W. Boiten (EATRIS/Lygature), S. Canham (ECRIN), M.L. Chiusano (EMBRC), W. Dastru (Euro-BioImaging), R. David (ERINHA), A. Laroquette (EMBRC), D. Longo (Euro-BioImaging), M.Th. Mayrhofer (BBMRI), M. Panagiotopoulou (ECRIN), A. Richard (ERINHA) (2021). The EOSC-Life WP4 toolbox: Update of the categorisation system. https://docs.google.com/document/d/13cVXp2Dyntpyg1r3md7-YDlITEI0mKfX/edit

5. R. David, C. Ohmann, J.W. Boiten, S.Canham, M. Panagiotopoulou, M.T. Mayrhofer, D. Longo, F. Bietrix, M.L. Chiusano, A. Laroquette, A. Richard, M. Cano-Abadia, W. Dastru, P.E. Verde (2021). EOSC-Life pilot study on the intercalibration of a categorisation system for FAIRer digital objects related to sensitive data in the life sciences. Poster for RDA's 18th Plenary Session. Zenodo. https://doi.org/10.5281/zenodo.5729437

6. C. Ohmann (ECRIN, pilot study coordinator), M. Cano Abadia (BBMRI), F. Bietrix (EATRIS), J.-W. Boiten (EATRIS/Lygature), S. Canham (ECRIN), M.L. Chiusano (EMBRC), W. Dastru (Euro-BioImaging), R. David (ERINHA), A. Laroquette (EMBRC), D. Longo (Euro-BioImaging), M.Th. Mayrhofer (BBMRI), M. Panagiotopoulou (ECRIN), A. Richard (ERINHA) (2021). EOSC-Life WP4 Toolbox: Update of the categorisation system (version 3). https://zenodo.org/record/5507324#.YbMN9L3MKUk

S5: Statistical analysis of the number of resources where only one tag was used per category

Statistical Analysis: Nature Scientific Reports

Pablo Emilio Verde

13 Juni 2022

Contents

1 Data setup for analysis **2**

2 Simple statistical analysis **2**

3 Data setup for statistical modeling **3**

4 Logistic regression **4**

4.1 Model diagnostics 5

4.2 Testing and adjusting by overdispersion in logistic regression 6

4.2.1 Testing for overdispersion 6

4.2.2 Adjusting for over-dispersion 8

1 Data setup for analysis

I transpose the table only for visualization…

```
col.names = c("Resource type",  "Research field",   "Data type",
              "Stage in data sharing lifecycle",
              "Geographical scope", "Specific topics")

row.names = c("Pilot study 1", "Pilot study 2", "Pilot study 3")

tab = matrix(c(62,  75, 68, 65, 99, 26,
               85,  53, 60, 110,    110,    45,
               80,  54, 79, 109,    108,    61),
             byrow = TRUE, nrow =3,
             dimnames = list(row.names, col.names))


kable(t(tab), digits = getOption("digits"), caption = "Contingency table.")%>%
  kable_styling(latex_options = "HOLD_position")
```

Table 1: *Contingency table.*

	Pilot study 1	Pilot study 2	Pilot study 3
Resource type	62	85	80
Research field	75	53	54
Data type	68	60	79
Stage in data sharing lifecycle	65	110	109
Geographical scope	99	110	108
Specific topics	26	45	61

2 Simple statistical analysis

Results of a statistical analysis by applying a χ^2 test for contingency tables.

```
# Simple statistical analysis ...

chisq.test(tab)
```

```
##
##  Pearson's Chi-squared test
##
## data:  tab
## X-squared = 30.779, df = 10, p-value = 0.0006381
```

3 Data setup for statistical modeling

```
col.names = c("Resource type",  "Research field",   "Data type",
              "Stage in data sharing lifecycle",
              "Geographical scope", "Specific topics")

dat.wide = data.frame(pilot = c(1:3),
           cat.1 = c(62,85,80),
           cat.2 = c(75,53,54),
           cat.3 = c(68,60,79),
           cat.4 = c(65,110,109),
           cat.5 = c(99, 110, 108),
           cat.6 = c(26, 45, 61))

dat.long = pivot_longer(dat.wide, cols = starts_with("cat"),
    names_to = "category",
    values_to = "count")

dat.long$n = 110
```

4 Logistic regression

I consider a Binomial outcome, where each cell in the table is

$$y \sim \text{Biomial}(p_i,\, n = 110).$$

The model is

$$\text{logit}(p_i) = \beta_0 + \text{effect for pilot 1,2,3} + \text{effect of category}$$

I compare: pilot 1 vs pilot 2 and pilot 1 vs pilot 3:

```
model.1 = glm(cbind(count, n-count) ~ factor(pilot) + category,
              family = binomial(link="logit"),
                data = dat.long)

summary(model.1)
```

```
##
## Call:
## glm(formula = cbind(count, n - count) ~ factor(pilot) + category,
##     family = binomial(link = "logit"), data = dat.long)
##
## Deviance Residuals:
##     Min       1Q   Median       3Q      Max
## -5.1092  -1.6510  -0.0107   1.9222   5.3774
##
## Coefficients:
##                 Estimate Std. Error z value Pr(>|z|)
## (Intercept)       0.3588     0.1384   2.592 0.009544 **
## factor(pilot)2    0.5532     0.1285   4.304 1.68e-05 ***
## factor(pilot)3    0.8019     0.1314   6.104 1.03e-09 ***
## categorycat.2    -0.5992     0.1646  -3.641 0.000272 ***
## categorycat.3    -0.2768     0.1667  -1.660 0.096848 .
## categorycat.4     1.0508     0.2004   5.245 1.57e-07 ***
## categorycat.5     2.4366     0.3083   7.904 2.70e-15 ***
## categorycat.6    -1.2284     0.1659  -7.403 1.33e-13 ***
## ---
## Signif. codes:  0 '***' 0.001 '**' 0.01 '*' 0.05 '.' 0.1 ' ' 1
##
## (Dispersion parameter for binomial family taken to be 1)
##
##     Null deviance: 537.77  on 17  degrees of freedom
## Residual deviance: 138.75  on 10  degrees of freedom
## AIC: 229.1
##
## Number of Fisher Scoring iterations: 5
```

4.1 Model diagnostics

The residual analysis shows that we do not have large residuals that would suggest outliers.

```
library(statmod)
qres = qresid(model.1)
par(mfrow = c(1,2))

qqnorm(qres)
qqline(qres)

scatter.smooth(qres~fitted(model.1), las = 1,
               main="Residuals vs. Fitted",
               xlab= "Fitted value", ylab="Quantile residual"
               )
```

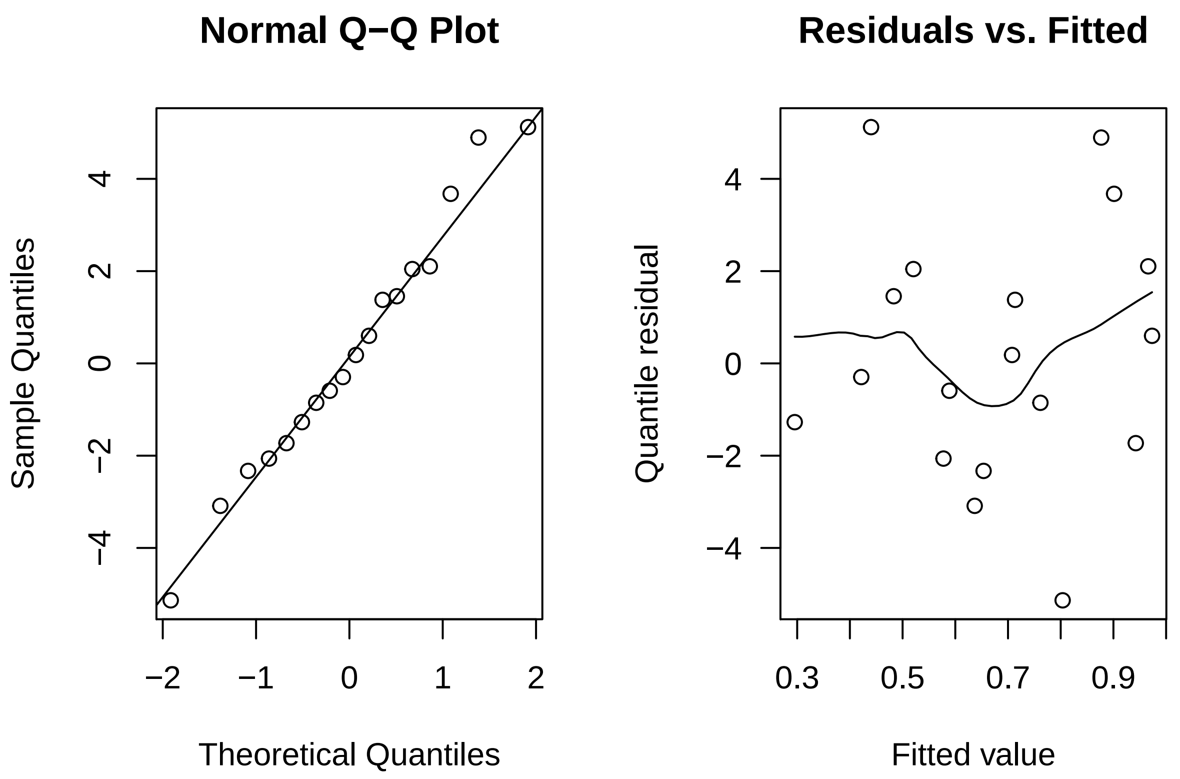


```
par(mfrow = c(1,1))
```

4.2 Testing and adjusting by overdispersion in logistic regression

4.2.1 Testing for overdispersion

We can check over-dispersion by dividing the residual deviance with the residual degrees of freedom of our binomial model:

$$\phi = \frac{\text{Residual deviance}}{\text{Residual df}},$$

If ϕ is close to 1, then we do not have over-dispersion, but for our data we have:

```
deviance(model.1)/df.residual(model.1)
```

```
## [1] 13.8747
```

This is a test for over-dispersion based on "Pearson residuals":

```
library(DHARMa)

testDispersion(model.1, type = "PearsonChisq", alternative = "greater")
```

```
##
##  Parametric dispersion test via mean Pearson-chisq statistic
##
## data:  model.1
## dispersion = 12.209, df = 10, p-value < 2.2e-16
## alternative hypothesis: greater
```

This is a test using simulations and the bootstrap method:

```
testDispersion(model.1,  alternative = "greater")
```

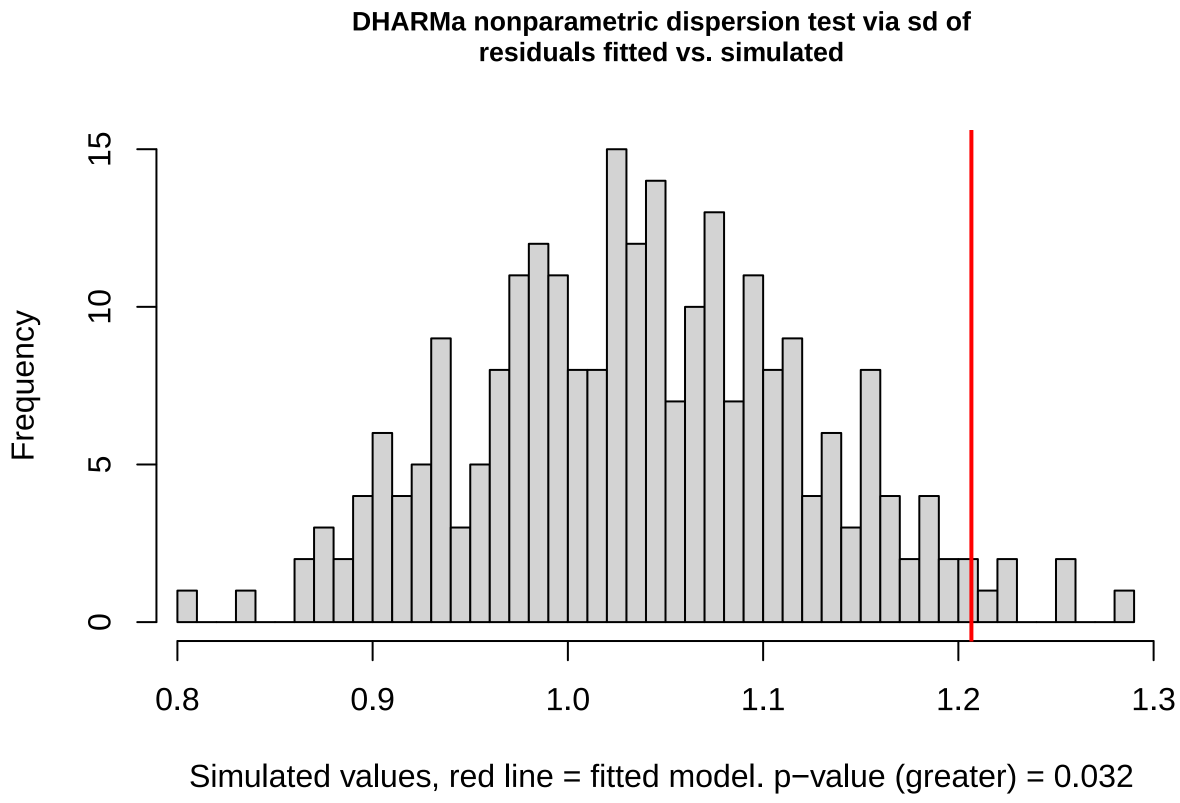


```
##
##  DHARMa nonparametric dispersion test via sd of residuals fitted vs.
##  simulated
##
## data:  simulationOutput
## dispersion = 1.1627, p-value = 0.032
## alternative hypothesis: greater
```

4.2.2 Adjusting for over-dispersion

```
model.1.quasi = glm(cbind(count, n-count) ~ factor(pilot) + category,
              family = quasibinomial(link="logit"),
                data = dat.long)

summary(model.1.quasi)
```

```
##
## Call:
## glm(formula = cbind(count, n - count) ~ factor(pilot) + category,
##     family = quasibinomial(link = "logit"), data = dat.long)
##
## Deviance Residuals:
##     Min       1Q   Median       3Q      Max
## -5.1092  -1.6510  -0.0107   1.9222   5.3774
##
## Coefficients:
##                 Estimate Std. Error t value Pr(>|t|)
## (Intercept)       0.3588     0.4837   0.742   0.4753
## factor(pilot)2    0.5532     0.4491   1.232   0.2463
## factor(pilot)3    0.8019     0.4590   1.747   0.1112
## categorycat.2    -0.5992     0.5752  -1.042   0.3220
## categorycat.3    -0.2768     0.5825  -0.475   0.6449
## categorycat.4     1.0508     0.7001   1.501   0.1643
## categorycat.5     2.4366     1.0772   2.262   0.0472 *
## categorycat.6    -1.2284     0.5798  -2.119   0.0602 .
## ---
## Signif. codes:  0 '***' 0.001 '**' 0.01 '*' 0.05 '.' 0.1 ' ' 1
##
## (Dispersion parameter for quasibinomial family taken to be 12.20959)
##
##     Null deviance: 537.77  on 17  degrees of freedom
## Residual deviance: 138.75  on 10  degrees of freedom
## AIC: NA
##
## Number of Fisher Scoring iterations: 5
```

Comments:

- After adjustment for over-dispersion the p-values are not significant for pilot 1 vs pilot 3.
- However, the confidence interval show a clear trend for pilot 1 vs. pilot 3.
- We can see the change in the lower bound of the confidence interval form 95% to 90%:

```
# 95% Confidence intervals for the parameters of the logistic regression
# after adjustment for over-dispersion:

confint(model.1.quasi, level = 0.95)[1:3,]
```

```
## Waiting for profiling to be done...

##                       2.5 %   97.5 %
## (Intercept)    -0.57536442 1.344764
## factor(pilot)2 -0.32067816 1.448551
## factor(pilot)3 -0.08614106 1.722741
```

```
# 90% Confidence intervals for the parameters of the logistic regression
# after adjustment for over-dispersion:

confint(model.1.quasi, level = 0.90)[1:3,]
```

```
## Waiting for profiling to be done...

##                         5 %     95 %
## (Intercept)    -0.42581577 1.179649
## factor(pilot)2 -0.18057044 1.302037
## factor(pilot)3  0.05558279 1.571266
```

S6: Toolbox demonstrator: Software development, implementation, and evaluation

(See also: Boiten, J.W. et al: EOSC-LIFE WP4 TOOLBOX: Toolbox for sharing of sensitive data - a concept description. Zenodo. https://doi.org/10.5281/zenodo.4483694)

For the development of the toolbox, a Technical Subcommittee was formed within EOSC-Life to explore technical options for the implementation of the toolbox, providing the user interface, searching, and presenting resources linked to sensitive data. As a first step, functional and non-functional as well as other metadata requirements were specified. Technical implementations of existing solutions and approaches potentially relevant for the design of the toolbox were evaluated and three different proposals for the technical implementation of the toolbox were explored: a fully customised solution, a partly customised solution with integration of external programs and a partly customised solution without full computer support of the tagging process. In addition, the costs and resources needed for the implementation of the proposed solutions were estimated. Finally, the approach providing a fully customised solution was chosen to be implemented by a developer from ECRIN for the following reasons: High flexibility and scalability, all requirements can be fulfilled, highest level of customization and modularity of the system.

The stacks used for software development are listed in table 1:

Core front-end development stack	Typescript (v. 4.0.2) - programming language<br>Angular (v.11) - the core technology / framework<br>Bootstrap (v. 4.6) - pages grid system, overall style, and design<br>Material design (v.11) - user interface, pages elements
The core back-end development stack	Python (v. 3.8) - programming language<br>Django (v. 3.0.2) - the core technology / framework<br>Django smart selects (v.1.5) - select boxes for the tagging system<br>Database SQLite (for development and testing purposes)<br>PostgreSQL (v.13) - production database.

Table 1: Development stacks for the toolbox demonstrator

The toolbox demonstrator was developed according to specified software requirements, with the software tools listed in table 1. The source code/repository used in front- and back-end development is publicly accessible (https://github.com/ecrin-github/toolbox-users-portal,https://github.com/ecrin-github/toolbox-tagging-demo) as well as the full list of packages used in the front-end development (https://github.com/ecrin-github/toolbox-users-portal/blob/main/package.json).

The 109 resources attached with the tags from pilot study 3 were used as the initial content for the toolbox demonstrator (0ne with missing data). The toolbox demonstrator is publicly available via the link: https://tsdo.ecrin-rms.org/. The tool allows pre-filtering of resources linked to sensitive data with free text in the title, by DOI or through authors. Further filtering is possible with respect to item type

(e.g., journal article, webinar, report, software) and selection of any of the pre-listed tags from the different categories of system version 3. The search result can be saved as PDF or JSON. The figure shows a screenshot from the toolbox demonstrator.

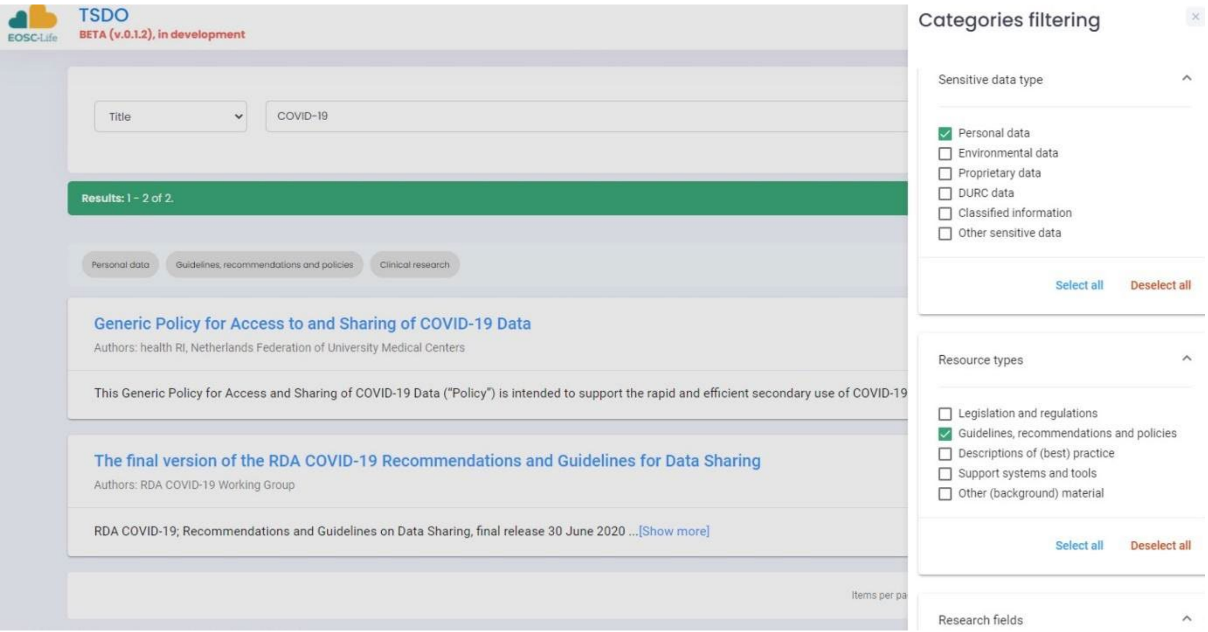

Figure: **Screenshot from the toolbox demonstrator**

A preliminary testing of the toolbox by untrained users was initiated and performed, involving 6 untrained users from 3 participating research infrastructures. The testers were asked to explore the toolbox and thereafter to enter a questionnaire. The results have been summarised in table 2.

**Question**	**Untrained user**					
	**A**	**B**	**C**	**D**	**E**	**F***
The toolbox is of interest for me	Agree	Agree	Strongly agree	Strongly agree	Agree	*Disagree*
The search capabilities to find sources about sensitive data are adequate	Agree	Agree	Agree	Agree	Agree	*Agree*
The handling of the toolbox is user-friendly	Agree	Agree	Neutral	Agree	Agree	*Strongly agree*
The output of the toolbox is presented in a useful and clear format	Agree	Neutral	Neutral	Strongly agree	Agree	*Strongly agree*
The toolbox has fast response times	Agree	Strongly agree	Stringly agree	Strongly agree	Agree	*Strongly agree*
The toolbox is useful in finding resources about sensitive data	Agree	Agree	Strongly agree	Agree	Agree	*Neutral*

Table 2: **Evaluation of user-friendliness by untrained users**
*User E had no experience with sensitive data

In summary, the response on the user-friendliness was positive with a majority agreeing to the statement “The handling of the toolbox is user-friendly”, “The search capabilities to find sources about sensitive data are adequate” and “The toolbox has fast response times”. All testers except the one without experience with sensitive data, agreed or strongly agreed to “The toolbox is of interest for me” and “The toolbox is useful in finding resources about sensitive data”. Nevertheless, room for improvement was indicated. This must be explored in a larger study on usability and user satisfaction, which is currently under planning.
